# The effectiveness, cost-effectiveness and experiences of interventions to reduce suicidality for autistic people: A scoping review

**DOI:** 10.1177/13623613251376208

**Published:** 2025-09-28

**Authors:** Noreen Orr, Liz Shaw, Simon Briscoe, Hassanat M. Lawal, Clara Martin-Pintado, Malcolm Turner, Jo Thompson Coon, Ruth Garside, G. J. Melendez-Torres

**Affiliations:** 1 University of Exeter, UK; 2 University of Exeter Medical School, UK

**Keywords:** autism, interventions, scoping review, screening, suicidality

## Abstract

Autistic people and people with elevated autistic traits are at a higher risk of suicidality (suicidal ideation, suicide plans, suicide attempts) than the general population, with over a third of autistic and possibly autistic people experiencing suicidal ideation, suicidal attempts and/or behaviour. The high prevalence of suicidality has been associated with lack of support and interventions to meet the specific needs of autistic people. This scoping review aimed to better understand the quantity and nature of existing primary research evaluating interventions to support autistic people experiencing suicidality, to inform the commissioning of future primary research. Twenty-seven studies were included: 18 focused on evaluating or developing interventions to reduce suicidality, and nine on evaluating/developing screening procedures to identify autistic people potentially at risk of dying by suicide. Findings suggest researchers are adapting and testing interventions to reduce suicidality in partnership with autistic people, but there is still work needed to enable autistic people to communicate suicidal thoughts and behaviours and develop clinician knowledge and understanding. While the number of completed studies using robust methods such as randomised controlled trials was small, this review indicates a nascent body of research evidence on interventions to reduce suicidality in the autistic population.

**Lay abstract:**

Autistic, or potentially autistic, people are at higher risk of experiencing suicidality than the general population. This has been linked to a lack of support and treatments that meet the specific needs of autistic people. This scoping review brings together research developing or evaluating strategies that aim to reduce the risk of autistic people dying by suicide. We reviewed 27 studies and found that there is a small but growing number of research projects that involve autistic people to develop treatments to reduce suicidality. For example, we found a study that has adapted and tested safety planning for autistic people. Other research has been testing tools that identify and assess suicidality and understanding healthcare professionals’ perspectives on assessing suicidality. More work is needed to develop training for professionals and on adapting assessment tools so that autistic people find it easier to talk about suicidal thoughts. Future research should also aim to be inclusive of the autistic population and ensure gender and cultural diversity in those that participate in research projects. Larger trials will be needed in the future to investigate the effectiveness of treatments for autistic people and build on existing evidence.

## Introduction

Autistic people and people with elevated autistic traits are at a higher risk of suicidality (suicidal ideation, suicide plans, suicide attempts) than the general population (Newell et al., 2023). A clinical cohort study of adults newly diagnosed with Asperger syndrome found that 66% reported suicidal ideation, nine times higher than the general population, and 35% reported plans or attempts at suicide (Cassidy et al., 2014). A matched case–cohort study showed autistic people are seven times more likely to attempt suicide than the general population, with autistic women twice as likely to attempt suicide as autistic men (Hirvikoski et al., 2020). Large cohort studies in Sweden and Denmark reported a 2.56-fold and a 3.83-fold increase in death by suicide for autistic people compared to the general population from 1987 to 2009 and from 1995 to 2016, respectively (Hirvikoski et al., 2016; Kõlves et al., 2021). A proportion of those with elevated autistic traits remain undiagnosed (Richards et al., 2019), and a recent study highlighted that those who died by suicide with undiagnosed possible autism were significantly higher than in the general population (41.4%; Cassidy et al., 2022).

In the United Kingdom, the National Health Service (NHS, 2019) Long Term Plan and Building the Right Support committed to increase the availability and accessibility of community mental health support, including crisis support, for autistic people to reduce the need for inpatient mental health care and ‘preventable deaths’ (NHS England, 2015). Despite these commitments, there is a lack of evidence on effective interventions to treat suicidality, with autistic people reporting that they receive interventions designed for other groups and inappropriate for their needs (Camm-Crosbie et al., 2019; Cassidy et al., 2021; Research ISfA, 2021). Adapting interventions to meet the needs of autistic people was one of the autism community’s top 10 priorities for future suicide research, identified in the International Research Priority Setting Exercise (2016–2019) and conducted in association with the James Lind Alliance (Cassidy et al., 2021).

The Department of Health and Social Care (DHSC, 2023) identified autistic people as a priority group in the National Suicide Prevention Strategy for England and committed to building the evidence on preventing suicidality to develop evidence-based policy and guidance. DHSC, with support from NHS England, commissioned the University of Exeter Policy Research Programme (PRP) Evidence Review Facility to undertake a scoping review of the peer-reviewed academic literature on interventions for autistic people experiencing suicidality to inform a decision on commissioning primary research to test interventions with autistic people, in part fulfilment of the National Suicide Prevention Strategy (DHSC, 2023). Thus, this review aims to better understand the quantity and nature of existing primary research evaluating interventions to support autistic people experiencing suicidality.

## Method

The methods were informed by the scoping review methodology of Arksey and O’Malley (2005) and the methodological enhancements of Levac et al. (2010). This scoping review is reported in accordance with PRISMA-Scr guidance (Tricco et al., 2018; see Supplementary File 1) and the protocol was registered on Zenodo (Orr et al., 2024).

### Identifying the research question

The SPIDER framework (Cooke et al., 2012) was used to formulate the question: What is the quantity, range and nature of studies on the effectiveness, cost-effectiveness and experiences of interventions to reduce suicidality for autistic people?

### Identifying the relevant studies

Bibliographic database search strategies included both free-text searching and indexing terms and combined search terms for autism with those for suicidality or self-harm. An example search strategy for MEDLINE can be found in Supplementary File 2. Bibliographic databases searched were: CENTRAL (Cochrane Library); CINAHL (EBSCO); Conference Proceedings Citation Index–Science (Web of Science); EconLit (EBSCO); MEDLINE (Ovid); and PsycInfo (Ovid). No date limits were applied except for the Conference Proceedings Citation Index (limited to the last 3 years to identify abstracts on research not yet published as journal articles).

We checked reference lists of studies meeting review-inclusion criteria, and reviewed reference lists of relevant reviews. We searched topically relevant websites by searching lists of publications and using search terms such as autis* and suicid* (see Supplementary File 3). We searched for unpublished trials via ClinicalTrials.gov and the World Health Organisation clinical trials registry (ICTRP) and contacted expert researchers for published/unpublished studies meeting the inclusion criteria.

### Study selection

We exported search results to Endnote 21 and de-duplicated these. To determine the clarity of the inclusion and exclusion criteria, three reviewers applied them to a sample (n = 100) of search results (L.S., N.O., H.L.). We discussed decisions to ensure consistent application of criteria and revised where necessary. Once finalised, two reviewers independently applied the revised eligibility criteria (see Table 1) to the title and abstract of each identified citation (L.S., N.O., H.L.), with disagreements resolved through discussion with a third reviewer as required. Full texts were assessed in the same way.

**Table 1.** Review question and inclusion criteria.

SPIDER term	Scoping review
Sample	Autistic people (adults/children/young people) with a diagnosis of autism/Asperger syndrome. People (adults, children and young people) who are possibly autistic (i.e. individuals scoring highly on measures of autistic traits but without a diagnosis).
Phenomenon of interest	Autism spectrum disorder (ASD) is defined as a disorder characterised by marked impairments in social interaction and communication accompanied by a pattern of repetitive, stereotyped behaviours and activities. Developmental delays in social interaction and language surface prior to age 3 years (ICD-10, n.d.). ASD can be co-diagnosed with certain genetic conditions. Suicidality: any suicidal thoughts and behaviours, such as suicidal ideation, suicidal intent and attempted suicide that, however without intervention, place someone at high risk for mental health crisis or suicide (Newell et al., 2023). Self-harming behaviours that were initially included as self-harm predicts suicidality in autistic people (Cassidy et al., 2018). However, a large number of studies were identified, which focused on reducing self-harm but with no clear evidence of suicidal intent. To keep the body of included evidence in line with the aims of this review, we excluded studies where suicidal risk or intent was not measured as an outcome.
Intervention	Any intervention: Intended to reduce suicidality in autistic people. This includes assessment-related interventions evaluating strategies to identify autistic people with suicidality. Evaluated for feasibility and acceptability in reducing suicidality in autistic people (includes implementation) intended to reduce suicidality evaluated for cost-effectiveness. Experiences of patients and the people who support them of interventions to reduce suicidality (e.g. process evaluations). Attitudes/perceptions of patients and people who support them on the design of interventions to reduce suicidality.
Design	Randomised controlled trials. Controlled trials. Prospective and retrospective cohort studies. Before and after studies. Modelling studies. Qualitative studies/process evaluations (based on interviews, focus groups and survey data). Feasibility studies (including those with no comparator). Research protocols. Conference abstracts.
Evaluation	Studies: Where outcome measure is a reduction in attempted suicides, completed suicides or suicidal thoughts and behaviour in autistic people. That explore experiences of interventions to reduce suicidality in autistic people. That explore attitudes and perceptions on design of interventions to reduce suicidality in autistic people.
Research type	Primary quantitative, qualitative and mixed-methods studies.

ICD-10: International Classification of Diseases, v.10.

### Charting the data

We developed a charting form using Microsoft Excel and piloted (L.S., N.O.) this on four articles. The revised form was used to collect information about population characteristics, interventions evaluated, study methods and outcomes (see Supplementary File 4). Although critical appraisal does not generally form part of the scoping study remit, we used the Mixed-Methods Appraisal Tool (MMAT; Hong et al., 2018) to provide a brief overview of the quality of key features of each included primary study. Both data extraction and quality appraisal were performed by one reviewer and checked by a second, with disagreements settled through discussion.

### Consultation

Alongside representatives from the DHSC, we also consulted members of PERSPEX, the patient and public involvement group who work with the Exeter PRP Evidence Review Facility. Four members of PERSPEX identified as neurodivergent (including autism) or as caring for an individual who was neurodivergent. The method of engaging with these stakeholder groups and impact on the review process is summarised in Supplementary File 5.

### Collation, summarising and reporting the results

We present a descriptive summary of the included studies, which summarises key features of the evidence: characteristics of studies, study design and quality and characteristics of the participants and settings. We then provide a more detailed narrative description of the phenomenon of interest evaluated in each study. Studies were categorised according to whether they focused on evaluating/developing interventions to reduce suicidality or on screening procedures intending to identify individuals at risk of dying by suicide. Studies of interventions were clustered according to the type of intervention being evaluated. Studies evaluating screening practices were separated into three groups: (1) those evaluating the efficacy of the Ask Suicide Screening Questions (ASQ; Horowitz et al., 2012); (2) those evaluating other types of universal screening measures for use in a population with autism; and (3) those exploring experiences of risk screening and/or management practices.

## Results

**Figure 1.**
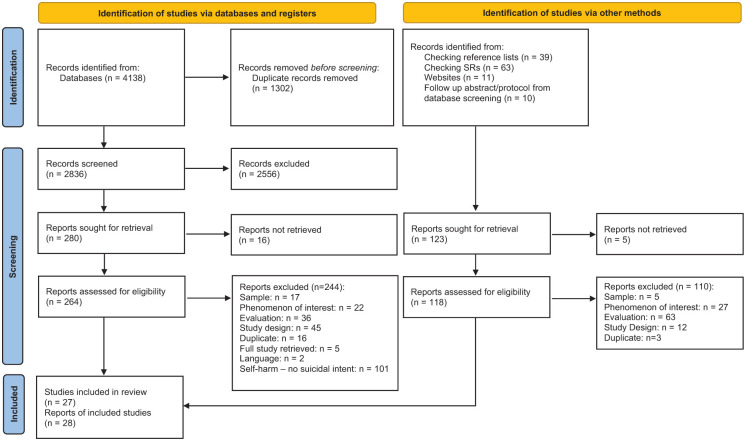
PRISMA diagram.

Bibliographic database searches identified 2836 unique records. We excluded 2556 records at title and abstract screening, leaving 280 to screen at full text. A further 123 records were identified via alternative search methods. The full texts of 403 records were sought for retrieval. Of the 382 full texts that could be retrieved, 355 were excluded (see Figure 1). We included 27 studies (reported in 28 articles) in this scoping review.

### Study characteristics

Twenty-seven studies (28 articles) were included: one study published two articles (Goodwin et al., 2024; Rodgers et al., 2024). Of the 27 included studies (28 articles), 16 were published as journal articles, including one pre-print (Goodwin et al., 2024). Six records were abstracts and six were trial registry items (i.e. ongoing trials that have not yet published results). Date of publication ranged from 2002 to 2024, with 16 studies published since 2022. Full details of the included studies can be found in Supplementary File 6.

### Setting

Studies were primarily from high-income, western countries: United States (n = 14), France (n = 3), Australia (n = 3), United Kingdom (n = 3), Netherlands (n = 2), Canada (n = 1) and Pakistan (n = 1). Twenty studies reported links with specific service settings: hospitals (n = 12), including adult or child psychiatry departments (n = 2), specialist ASD centres (*n* = 1), rehabilitation centres (n = 1), outpatient mental health centres (n = 1), paediatric psychiatric emergency departments (n = 6) and emergency departments (n = 2) Four studies included participants outside of hospital settings: health centres (n = 3) and psychology clinics (n = 1). Three studies were conducted in, or recruited participants from, a variety of settings and seven studies (eight articles) did not report a specific setting.

### Study design

Eight studies were randomised controlled trials (RCTs; Ameis, 2021; Huntjens et al., 2024; Kalb, 2022; Roubinov, 2022; Santomauro et al., 2016; Schiltz et al., 2018; Shafique & Chaudhry, 2023; Weiner, 2021), which included one pilot RCT, four pending completion (recorded as trial registry items) and one website abstract reported an ongoing pilot trial. One study used qualitative research to inform the development of a safety planning intervention, which was then evaluated as a feasibility RCT using mixed methods (Goodwin et al., 2024; Rodgers et al., 2024). Of the four completed studies with an RCT element, sample sizes ranged from 23 (Santomauro et al., 2016) to 123 (Huntjens et al., 2024).

Twelve studies used other quantitative methods: cross-sectional (n = 7), retrospective chart reviews (n = 4) and before and after study designs (n = 1; Cashin et al., 2013). Two studies were part of wider service improvement projects (Cervantes et al., 2024b; Kalb et al., 2022). Three studies used a qualitative design (Cervantes et al., 2024a, 2024c; Goodwin et al., 2024), collecting data using semi-structured interviews and/or online focus groups and surveys. Five other studies also used mixed methods.

### Sample

Fourteen studies included only autistic people, eight of which included only adults (Ameis, 2021; Bal et al., 2024; Bemmouna et al., 2022; Cassidy et al., 2020; Huntjens et al., 2024; Lobregt-van Buuren, 2022; Rodgers et al., 2024; Weiner, 2021), and six included only children and/or young people (Cashin et al., 2013; Kalari et al., 2022; Santomauro et al., 2016; Schiltz et al., 2018; Vasa et al., 2017; Zoha et al., 2022). Three studies focused on caregivers (Kalb, 2022; Kalb et al., 2022; Rybczynski et al., 2022) and four on clinicians (Cervantes 2023; Cervantes et al., 2023; Jager-Hyman et al., 2020; Zoha et al., 2022). Six studies included a mix of participants: young people and parents/carers (n = 2; Cervantes et al., 2024b; Horowitz et al., 2018); and autistic people, caregivers, clinicians and/or healthcare providers/leaders (n = 4). Seventeen studies required a formal diagnosis of an autistic spectrum disorder; two did not require a formal diagnosis (Consoli et al., 2013; Horowitz et al., 2018), six studies did not report this information (Cervantes 2023; Kalb, 2022; Rybczynski et al., 2022; Shafique & Chaudhry, 2023; Vasa et al., 2017; Weiner, 2021), with this data not applicable to the remaining three studies (Cervantes et al., 2023; Jager-Hyman et al., 2020; Zoha et al., 2022), as their focus was on clinicians.

The total number of participants in the quantitative studies ranged from 4 (Consoli et al., 2013) to 1665 (Roubinov, 2022), and from 14 (Cervantes et al., 2024a) to 46 in the qualitative studies (Goodwin et al., 2024) and from 26 (Bal et al., 2024) to 371 in mixed methods studies (Cassidy et al., 2020). The median sample size across all study designs was 123 participants.

The number of autistic people reported in 18 studies ranged from 4 (Consoli et al., 2013) to 699 (Rybczynski et al., 2022), with the percentage of female participants ranging from 8 (Schiltz et al., 2018) to 70 (Goodwin et al., 2024). Data from six studies indicated that the number of carers ranged from 5 (Goodwin et al., 2024) to 404 (Kalb et al., 2022), with carer involvement primarily from women. Data from six studies indicated that the number of clinicians per study ranged from 3 (Cervantes et al., 2024a) to 100 (Cassidy et al., 2020), with the percentage of female participants ranging from 56 (Goodwin et al., 2024) to 100 (Cassidy et al., 2020; Cervantes et al., 2024a) and one was a conference abstract and did not report this information (Zoha et al., 2022).

### Critical appraisal

The sixteen completed studies (17 articles) published as journal articles were quality appraised using the MMAT screening tool (Hong et al., 2018; see Supplementary File 7).

#### Qualitative studies

Of the four articles appraised using the qualitative MMAT items, three scored positively across all five criteria (Cassidy et al., 2020; Cervantes et al., 2024c; Goodwin et al., 2024), and one did not provide sufficient participant quotes to evidence the study findings (Cervantes et al., 2024a).

#### Randomised controlled trials

Of the three studies appraised using criteria for RCTs, all scored positively on items pertaining to having comparable groups at baseline and complete outcome data but had poor participant adherence to the intervention (Huntjens et al., 2024; Santomauro et al., 2016; Schiltz et al., 2018). Ratings could not be given for two studies on items relating to appropriate randomisation and blinding of outcome assessors (Santomauro et al., 2016; Schiltz et al., 2018).

#### Descriptive studies

None of the seven studies appraised using the MMAT for descriptive studies (Cashin, 2008; Cervantes et al., 2023, 2024b; Consoli et al., 2013; Jager-Hyman et al., 2020; Kalb et al., 2022; Rybczynski et al., 2022) scored positively across all five appraisal criteria. Two studies scored negatively on only gaining a representative sample (Cashin, 2008; Consoli et al., 2013) and only one study scored positively (Cervantes et al., 2024b). One other study only scored negatively on the item appraising risk of non-response bias (Cervantes et al., 2024b). Of the remaining four studies, two only scored positively on two items (Cervantes et al., 2023; Rybczynski et al., 2022) and two positively on three (Jager-Hyman et al., 2020; Kalb et al., 2022).

#### Mixed-methods studies

The three studies using mixed methods were appraised as poorly conducted (Bal et al., 2024; Bemmouna et al., 2022; Rodgers et al., 2024), with only one study scoring positively against one of the five criteria (Bal et al., 2024). This was due to poor reporting of qualitative methods (Bemmouna et al., 2022) and poor integration of quantitative and qualitative findings (Bal et al., 2024; Bemmouna et al., 2022; Rodgers et al., 2024).

#### Studies evaluating interventions

The intervention studies (n = 18) were grouped into six broad intervention categories: safety planning (n = 5), dialectical behaviour therapy (DBT; n = 4), cognitive behaviour therapy (CBT; n = 2), psychosocial therapy and other (n = 2), training (n = 3) and electroconvulsive therapy (ECT)/repetitive transcranial magnetic stimulation (rTMS) (n = 2). Further details of these are in Supplementary File 8.

### Safety planning

Four studies focused on interventions to support autistic adults (Bal et al., 2024; Goodwin et al., 2024; Rodgers et al., 2024), children and young people (Roubinov, 2022), and parents of autistic children to prepare plans to be used leading up to or during a crisis to reduce self-harm and the risk of dying by suicide (Kalb, 2022). One study focused on clinicians’ knowledge and confidence in using safety plans with autistic people (Jager-Hyman et al., 2020). Suicidality was the primary target for the interventions evaluated by all four studies. Three of the interventions were based on the structure of the original Stanley–Brown Safety Plan (Bal et al., 2024; Goodwin et al., 2024; Jager-Hyman et al., 2020; Rodgers et al., 2024), comprising a list of hierarchical steps, which are warning signs, internal coping strategies, social contacts and locations, family members or friends that may offer help, professionals or agencies to help and how to keep the environment safe (Stanley & Brown, 2012). Some of these elements were also present in the other two interventions such as warning signs (Roubinov, 2022) and connections to professional and lay resources (Kalb, 2022). The interventions were delivered via telehealth and all involved clinicians, licenced clinical therapists, health system leaders or support workers (Bal et al., 2024; Kalb, 2022; Rodgers et al., 2024). One intervention had a self-guided version (Bal et al., 2024) and in another, researchers were involved in its delivery (Rodgers et al., 2024). Adapting safety plans to meet the needs of autistic people was key to the Autism Adapted Safety Plans (AASP) intervention (Rodgers et al., 2024) and Stage One patient and public involvement (PPI) work involved co-developing a resource pack and clarifying the safety plan template to meet ‘autistic thinking’ and communication styles (Goodwin et al., 2024; Rodgers et al., 2024, p. 4). Support workers and researchers received training that had been co-designed with autistic people, including information on suicide and self-harm, working with autistic people and opportunities to practise the AASP (Rodgers et al., 2024). Similarly, the Emotional Support Plan involved autistic people, who were members of the study team, in co-developing the videos and other materials for the self-guided version of the intervention (Bal et al., 2024).

### Dialectical behaviour therapy

Four studies evaluated DBT interventions, which aimed to decrease emotion dysregulation and maladaptive coping, and improve depression, hopelessness, anxiety, alexithymia and the frequency and intensity of self-harm and suicidal ideation for autistic adults (Bemmouna et al., 2022; Huntjens et al., 2024; Lobregt-van Buuren, 2022; Weiner, 2021). Suicidality was the primary target in all four studies. Four modules of DBT skills – mindfulness, emotion regulation, distress tolerance and interpersonal effectiveness skills – were covered in two interventions across varying time periods – one was 18 weeks (Bemmouna et al., 2022) and another was 26 weeks (Huntjens et al., 2024). These interventions were based on four components: weekly individual therapy sessions, weekly skills training group sessions, access to telephone coaching with an individual therapist and weekly therapist consultations (Bemmouna et al., 2022; Huntjens et al., 2024). One trial in progress included a body-oriented DBT-skills training for possible impairments of interoceptive body awareness (Lobregt-van Buuren, 2022). Interventions were delivered by trained DBT clinical psychologists, therapists and counsellors. Two were delivered in outpatient settings (Bemmouna et al., 2022; Huntjens et al., 2024), one in both outpatient and inpatient settings (Lobregt-van Buuren, 2022), with setting not reported by Weiner (2021). Two interventions were adapted for autistic adults by addressing the therapy environment, social anxiety and ensuring that the delivery was modified to suit autistic communication styles (Huntjens et al., 2024). The therapy environment was kept as stable as possible in terms of room layout, schedule and facilitators (Bemmouna et al., 2022). Pre-treatment sessions were provided to prepare participants for the demands of the DBT intervention, its structure and goals and to support participation in group sessions, as well as support planning between sessions (Bemmouna et al., 2022; Huntjens et al., 2024). DBT manuals were modified, reducing text and simplifying explanations of certain DBT skills to make them more concrete and understandable and instructions for the exercises at the beginning of the skills training were ‘precise and unambiguous’ (Bemmouna et al., 2022; Huntjens et al., 2024).

### Cognitive behavioural therapy

Two RCTs evaluated the effects of CBT on self-reported symptoms of depression and on self-harm and suicidality in adolescents and young people (Santomauro et al., 2016; Shafique & Chaudhry, 2023). One trial in progress evaluates a manual-assisted psychological intervention, involving cultural adaptation for Pakistani young people. Suicidality is the primary target, but little details on the components of the intervention and its delivery are reported (Shafique & Chaudhry, 2023). The Exploring Depression Intervention comprised sessions exploring different ‘tools’ adolescents could use to manage symptoms of depression: self-awareness, physical, pleasure, thinking, social and relaxation tools. Suicidality was an outcome measured in this study. There were 10 sessions in which the tools were discussed, with young people reflecting on when they used the tools and how they felt before and after. The intervention was delivered in a university psychology clinic by two provisionally registered clinical psychologists and supervised by two senior clinical psychologists. The only adaptation for autism reported was that one home project for the young people required them to read an article describing the positive qualities of ASD (Santomauro et al., 2016).

### Psychosocial therapy and narrative therapy

Two interventions used narrative therapy and social skills to reduce stress-related problems and potentially reduce depression for adolescents with autism (Cashin et al., 2013; Schiltz et al., 2018). Young autistic people often present with ‘problem-saturated narratives . . . accompanied by high degrees of anxiety and depression’ (Cashin et al., 2013, p. 34). The externalisation techniques in narrative therapy may help them identify problem ‘form’ and turn it into a ‘concrete entity’ (Cashin, 2008). Following problem identification, the young person and therapist develop an action plan, and ‘co-author a new narrative’. Outside the five sessions of the intervention, the young person undertakes ‘out-of-session’ work in recording and illustrating the journey, with the visual element playing to the strength of those with autism. The focus of this intervention was reducing levels of hopelessness. a phenomenon highly correlated with suicidality. The second intervention, the 14 week Program for the Education and Enrichment of Relational Skills (PEERS^®^), was premised on the notion that increasing social skills and friendships would ameliorate depressive symptoms, and in turn suicidality, in young people with ASD (Schiltz et al., 2018). PEERS^®^ was based on an evidence-based parent-assisted social skills curriculum (Frankel & Myatt, 2003), but modifications were made to the curriculum and teaching methods to be more appropriate for young autistic people (Schiltz et al., 2018).

### Training

Three studies focused on training to improve quality of care for autistic young people by increasing clinician confidence in recognising and diagnosing ASD, screening for and identifying suicide risk, and managing and intervening on suicide risk (Cervantes et al., 2023); encouraging clinicians to implement routine suicide risk screening with autistic young people (Cervantes, 2023); and increasing trainee (e.g. child and adolescent psychiatry fellows and psychology interns) confidence in assessing and addressing suicidal thoughts and behaviours in young people presenting in hospital emergency departments (Zoha et al., 2022). The Safe Alternatives for Teens and Youths Acute (SAFETY-A) intervention involved two educational videos about SAFETY-A and a case simulation, which was completed by the trainees during a rotation on the emergency service of a hospital (Zoha et al., 2022). The training for the implementation of routine suicide risk screening with autistic young people is in progress, and educational materials will be developed for caregivers and clinicians and disseminated via webinars and other websites (Cervantes, 2023).

### Use of ECT/rTMS

Two studies evaluated electroconvulsive therapy (ECT) and rTMS to alleviate self-injurious behaviour and aggression, and reduce depression in children and young autistic people and PDD-NOS (pervasive developmental disorder-not otherwise specified; Ameis, 2021; Consoli et al., 2013). The extent to which the interventions evaluated targeted suicidality varied; in one study, only one participant demonstrated suicidal behaviour, which was the target of the intervention (Ameis, 2021), whereas suicidality was an outcome measure for all participants in the other (Consoli et al., 2013).

#### Studies evaluating suicide-screening procedures

Ten studies evaluated suicide risk screening procedures; five focused on the use of the ASQ, three evaluated the feasibility/useability of other universal screening tools for autistic people, and two studies explored broader attitudes towards screening practices to identify individuals at risk of suicide (Cervantes et al., 2024c; Jager-Hyman et al., 2020). Further details of these screening tools are detailed in Supplementary File 9.

### Ask suicide screening questions

Five studies reported in two journal articles (Cervantes et al., 2024b; Rybczynski et al., 2022) and three abstracts (Horowitz et al., 2018; Kalari et al., 2022; Vasa et al., 2017) focused on using the ASQ to identify suicide risk in children with autism. The average age of children being screened (for the three studies providing this information) ranged from 12.1 (Rybczynski et al., 2022) to 15 years (Horowitz et al., 2018). Four quantitative studies evaluated the effectiveness of the five-item ASQ in identifying youth at risk of suicide within the emergency department (Cervantes et al., 2024b; Kalari et al., 2022; Rybczynski et al., 2022; Vasa et al., 2017). One study explored the feasibility of using the ASQ in a medical setting (Horowitz et al., 2018) and one study described the implementation of a screening programme in an emergency department and factors influencing engagement (Rybczynski et al., 2022). Two studies compared the efficacy of the ASQ with other measures, including the Kiddie-Computerized Adaptive Test Suicide Scale (KCAT-SS; Cervantes et al., 2024b) and the Columbia-Suicide Severity Rating Scale (Rybczynski et al., 2022).

### Universal risk screening tools

Three studies evaluated the useability or feasibility of universal risk screening tools for autistic people (Cassidy et al., 2020; Cervantes et al., 2024a; Kalb et al., 2022). Each study evaluated the use of one different screening tool administered online, including the Suicidal Behaviours Questionnaire (revised; Cassidy et al., 2020) and the KCAT-SS (Cervantes et al., 2024a). These screening measures specifically aimed to assess suicide risk and differed significantly in length, from 4 to 64 items, respectively. The third study explored attitudes towards the non-suicide-specific Mental Health Crisis Assessment Scale (revised), intended to appraise the severity of 13 mental health-related externalising behaviours, which encompassed suicidal thoughts and behaviour, and parental self-efficacy in managing these (Kalb et al., 2022). The intended settings reported were an emergency department (Cervantes et al., 2024c) and outpatient psychiatric, behavioural or psychiatric clinic in ASD specialty centres (Kalb et al., 2022).

### Broader experiences of suicide risk screening

Two studies explored broader experiences and/or attitudes towards screening practices to identify autistic people at risk of suicide (Cervantes et al., 2024c; Jager-Hyman et al., 2020). The first analysed qualitative interviews with young autistic people, carers and autism specialist clinicians to develop recommendations for improving suicide risk screening and management within the emergency department (Cervantes et al., 2024c). The second used surveys to examine and compare clinician perspectives and experiences of suicide risk screening practices in autistic versus non-autistic populations (Jager-Hyman et al., 2020). This study also examined clinician experiences of using a Safety Planning Intervention.

## Discussion

This review presents an overview of the current research evidence evaluating interventions to support autistic people experiencing suicidality. It suggests that since the publication of the International Research Priority Setting Exercise on the priorities for future suicide research in 2021, some progress has been made in developing research projects that align with the needs of the autism community. Some researchers are adapting interventions for reducing suicidality in partnership with autistic people; for example, a pilot feasibility RCT of AASP to reduce self-harm and suicide for people with autism has been published (Rodgers et al., 2024). Other interventions adapted existing treatments used with other populations such as DBT (Bemmouna et al., 2022; Huntjens et al., 2024; Lobregt-van Buuren, 2022), narrative therapy (Cashin, 2008) and social skills training (Schiltz et al., 2018). Some of these adaptations focused on communicating clearly to suit ‘autistic thinking’ with modifications to DBT manuals and precise, unambiguous instructions for exercises and skills training sessions (Bemmouna et al., 2022; Huntjens et al., 2024). Only one study specifically mentioned adjustments to the sensory environment in terms of location and noise (Bemmouna et al., 2022). According to Brice et al. (2021), sensory environment is key, and a lack of adjustments to meet the needs of autistic people could contribute to their failure to complete therapy, which could have serious consequences.

Another research priority identified by the autism community was about understanding how autistic people present at services with suicidal thoughts and behaviours, how this is perceived by clinicians and how services could better support them (Cassidy et al., 2021). This review reports some nascent work on clinician perspectives on assessing and managing suicide risk in autistic people. Two surveys found clinicians can lack confidence and expertise in delivering care for autistic people presenting with suicidal thoughts and behaviours (Cervantes et al., 2023a; Jager-Hyman et al., 2020). There is clearly a need for developing and disseminating training for clinicians and Cervantes’s (2023) proposed research project aims to produce educational materials for clinicians to encourage the implementation of routine suicide risk screening with autistic young people. There is considerable scope for further work developing training for service providers in partnership with autistic people to ensure that they have the necessary knowledge and skills (Hedley et al., 2017). For autistic people, clinician knowledge and understanding are the most important adjustments that would improve care in a mental health setting (Brice et al., 2021).

Identifying and assessing suicidal thoughts and behaviours in autistic people was another of the autism community’s research priorities (Cassidy et al., 2021). One proposed future research area was to establish how suicidality assessment tools for the general population could be adapted for autistic people. This aligned with the recommendation by Cassidy et al. (2018) for adapting current tools to better conceptualise suicidality and its measurement in autism. This review highlights ongoing work on assessment-related interventions to identify and screen for autistic people with suicidality. Much of this focuses on using existing tools such as the SBQ-R (Cassidy et al., 2020), the K-CAT and the K-CAT-SS (Cervantes et al., 2024a) and the ASQ (Cervantes et al., 2024b; Kalari et al., 2022; Rybczynski et al., 2022; Vasa et al., 2017). There were examples of qualitative research to gain feedback from autistic people, carers and autism specialist clinicians on existing suicide assessment tools (Cassidy et al., 2020; Cervantes et al., 2024a). Findings revealed that language issues were common, with difficulties interpreting questions, understanding abstract questions, confusing phrases and vocabulary, and distinguishing between question options. This highlights that modifications are necessary and that there is still much to be done to enable autistic people to communicate suicidal thoughts and behaviours. In addition, the screening tool evidence base is primarily confined to emergency departments and future work should include non-emergency care settings if screening tools are to be community-wide ‘preventative’ tools.

### Strengths and limitations

Unlike systematic reviews, scoping reviews do not pool or synthesise the included evidence but rather ‘describe, categorise and catalog’ the evidence (Campbell et al., 2023). However, as part of the ‘Big Picture’ review family (Campbell et al., 2023, p. 6), scoping reviews address broad research questions and by identifying available evidence, can inform future policy and research decisions. This review follows best practice guidelines for scoping reviews and includes quality appraisal of the peer-reviewed publications. Robust searches identified trials in progress and other studies that were planned or in progress via abstracts and websites. It is helpful in an emerging research field to be aware of interventions being developed and evaluated to help prevent duplication of effort. However, some abstracts and trial registry items were sparse in details and important information on adaptations for autism were not reported. Given the stage of the research cycle, there were a limited number of completed studies using robust effectiveness methods, such as RCTs, evaluating the same or similar interventions or screening programmes/approaches, which precludes further synthesis to establish intervention effectiveness at this time. Furthermore, although we searched for cost-effectiveness studies, no interventions were evaluated for cost-effectiveness, again potentially reflecting the nascent nature of the field. Generally, the qualitative evidence was of better quality than the quantitative evidence, but there was poor integration of findings across quantitative and qualitative components within mixed-methods studies. There was little explanation of the theory base for many of the interventions, which likely reflects the observations that research has tended not to be ‘theoretically driven’ (Cassidy et al., 2020), and existing theories explaining suicidality in the general population have not yet been successfully adapted to the autistic population (Cassidy et al., 2018). Almost all of the studies were from high-income countries, reflecting autism research in general (Durkin et al., 2015). Calls to expand the global reach of autism research are pertinent to autism and suicidality, with the need to develop culturally appropriate interventions to reduce suicidality for autistic people in diverse communities around the globe (Rice & Lee, 2017).

Researchers have been working with service users including adults, young adults and children, and others in the autism community. However, in several studies, authors noted issues with their samples in terms of size (Bal et al., 2024; Bemmouna et al., 2022; Santomauro et al., 2016; Schiltz et al., 2018) and representativeness, particularly in relation to ethnicity. Those completing feasibility and acceptability studies recognised that future trials and studies would need to ensure ethnic and cultural diversity (Bemmouna et al., 2022; Rodgers et al., 2024). An autism diagnosis as an inclusion criterion may have impacted the ethnic diversity of study participants (Goodwin et al., 2024), reflecting structural racism in diagnostic practices (Giwa Onaiwu, 2020; Jones et al., 2020). Women and adolescent girls were represented in most of the published studies, but there is growing recognition that the differences in suicide risk between autistic men and women have implications for diagnosis and treatment of women and adolescent girls (Green et al., 2019; Hirvikoski et al., 2020). The interpretation of screening and assessment tools across genders is a case in point and Cassidy et al. (2020) noted that ‘future research on the measurement properties of adapted autism specific suicidality assessment tools should test for measurement invariance between autistic men and women’ (p. 3486). It is also important that future work recognises other genders, as more autistic people identify as non-binary compared to the general population (Peachey & Crane, 2024).

## Conclusion

This scoping review indicates that there is nascent body of research evidence on interventions to reduce suicidality in the autistic population. A range of interventions aiming to reduce suicidality have been developed and evaluated for leading up to and during a crisis, such as safety planning, to those more upstream interventions to reduce stress, anxiety, depression, and self-injurious behaviour. While progress has been made to address research priorities for the autism community in terms of reducing suicide risk, the body of existing evidence in this area is still limited. Hence, there is much scope for commissioning of future primary research for the design and evaluation of interventions and screening procedures to reduce suicidality in this population.

## Supplemental Material

sj-docx-1-aut-10.1177_13623613251376208 – Supplemental material for The effectiveness, cost-effectiveness and experiences of interventions to reduce suicidality for autistic people: A scoping review Supplemental material, sj-docx-1-aut-10.1177_13623613251376208 for The effectiveness, cost-effectiveness and experiences of interventions to reduce suicidality for autistic people: A scoping review by Noreen Orr, Liz Shaw, Simon Briscoe, Hassanat M. Lawal, Clara Martin-Pintado, Malcolm Turner, Jo Thompson Coon, Ruth Garside and G. J. Melendez-Torres in Autism
