# Supplementary material for: The effectiveness, cost-effectiveness and experiences of interventions to reduce suicidality for autistic people: A scoping review
Source: Autism. 2025 Sep 28;30(3):561–73. doi: 10.1177/13623613251376208 (PMC12923620; doi:10.1177/13623613251376208)
Supplement: sj-docx-1-aut-10.1177_13623613251376208 – Supplemental material for The effectiveness, cost-effectiveness and experiences of interventions to reduce suicidality for autistic people: A scoping review [file sj-docx-1-aut-10.1177_13623613251376208.docx]

**Supplementary Material**

File 1: PRISMA-Scr Checklist Statement p. 2

File 2: Search strategy for MEDLINE  p. 4

File 3: Websites searched p. 5

File 4: Data Extracted p. 6

File 5: Stakeholder consultation p. 7

File 6: Characteristics of Studies p. 8

File 7: Critical appraisal of included studies p. 28

File 8: Studies evaluating interventions p. 31

File 9: Studies evaluating suicide-screening procedures p. 46

**Supplementary File 1: PRISMA-Scr Checklist Statement**

| **Section** | **Item** | **PRISMA-ScR Checklist Item** | **Page** |
| --- | --- | --- | --- |
| **Title** | 1 | Identify the report as a scoping review. | 1 |
| Abstract  Structured summary | 2 | Provide a structured summary that includes (as applicable) background, objectives, eligibility criteria,  sources of evidence, charting methods, results, and conclusions that relate to the review questions  and objectives. | 2 |
| **Introduction** |  |  |  |
| Rationale | 3 | Describe the rationale for the review in the context of what is already known. Explain why the review  questions/objectives lend themselves to a scoping review approach. | 3 |
| Objectives | 4 | Provide an explicit statement of the questions and objectives being addressed with reference to their key elements (e.g., population or participants, concepts, and context) or other relevant key elements used to conceptualize the review questions and/or objectives. | 4 |
| **Methods** |  |  |  |
| Protocol and registration | 5 | Indicate whether a review protocol exists; state if and where it can be accessed (e.g., a Web address); and if available, provide registration information, including the registration number. | 3 |
| Eligibility criteria | 6 | Specify characteristics of the sources of evidence used as eligibility criteria (e.g., years considered, language, and publication status), and provide a rationale. | 4 |
| Information sources* | 7 | Describe all information sources in the search (e.g., databases with dates of coverage and contact with authors to identify additional sources), as well as the date the most recent search was executed. | 5 |
| Search | 8 | Present the full electronic search strategy for at least 1 database, including any limits used, such that it could be repeated. | SF 2: 3 |
| Selection of sources of evidence† | 9 | State the process for selecting sources of evidence (i.e., screening and eligibility) included in the scoping review. | 5 |
| Data charting process‡ | 10 | Describe the methods of charting data from the included sources of evidence (e.g., calibrated forms or forms that have been tested by the team before their use, and whether data charting was done independently or in duplicate) and any processes for obtaining and confirming data from investigators. | 5 |
| Data items | 11 | List and define all variables for which data were sought and any assumptions and simplifications made. | 5 |
| Critical appraisal of individual sources of  evidence§ | 12 | If done, provide a rationale for conducting a critical appraisal of included sources of evidence; describe the methods used and how this information was used in any data synthesis (if appropriate). | 5 |
| Summary measures | 13 | Not applicable for scoping reviews. | NA |
| Synthesis of results | 14 | Describe the methods of handling and summarizing the data that were charted. | 6 |
| Risk of bias across studies | 15 | Not applicable for scoping reviews. | NA |
| Additional analyses | 16 | Not applicable for scoping reviews. | NA |
| **Results** |  |  |  |
| Selection of sources of evidence | 17 | Give numbers of sources of evidence screened, assessed for eligibility, and included in the review, with reasons for exclusions at each stage, ideally using a flow diagram. | 6 |
| Characteristics of sources of evidence | 18 | For each source of evidence, present characteristics for which data were charted and provide the citations. | 6-8 |
| Critical appraisal within sources of evidence | 19 | If done, present data on critical appraisal of included sources of evidence (see item 12). | 8-9 |
| Results of individual sources of evidence | 20 | For each included source of evidence, present the relevant data that were charted that relate to the review questions and objectives. | 9-13 |
| Synthesis of results | 21 | Summarize and/or present the charting results as they relate to the review questions and objectives. | 9-13 |
| Risk of bias across studies | 22 | Not applicable for scoping reviews. | NA |
| Additional analyses | 23 | Not applicable for scoping reviews. | NA |
| **Discussion** |  |  |  |
| Summary of evidence | 24 | Summarize the main results (including an overview of concepts, themes, and types of evidence available), link to the review questions and objectives, and consider the relevance to key groups. | 13-4 |
| Limitations | 25 | Discuss the limitations of the scoping review process. | 14-5 |
| Conclusions | 26 | Provide a general interpretation of the results with respect to the review questions and objectives, as well as potential implications and/or next steps. | 16 |
| Funding | 27 | Describe sources of funding for the included sources of evidence, as well as sources of funding for the scoping review. Describe the role of the funders of the scoping review. | Title page |

**Supplementary File 2: Search Strategy for Medline**

Database: MEDLINE ALL
Host: Ovid
Issue: 1946 to May 10, 2024
Date Searched: 13/05/2024
Searcher: SB
Hits: 1527
Strategy:

1. (autis* or asperg* or "pervasive development* disorder*" or "high functioning" or asc or asd).tw.
2. exp Autism Spectrum Disorder/
3. Child Development Disorders, Pervasive/
4. or/1-3
5. (suicid* or parasuicid* or automutilation*).tw.
6. (self adj2 (harm* or injur*)).tw.
7. exp Self-Injurious Behavior/
8. or/5-7
9. 4 and 8

**Supplementary File 3: Websites searched**

- Ambitious about Autism <https://www.ambitiousaboutautism.org.uk/>
- Autism Alliance UK <https://www.autism-alliance.org.uk/>
- Autism Central <https://www.autismcentral.org.uk/>
- Autism Centre of Excellence <https://www.autismcentreofexcellence.org/>
- Autistica <https://www.autistica.org.uk/>
- Daisy chain <https://daisychainproject.co.uk/>
- MQ Research <https://www.mqmentalhealth.org/home/>
- National Autistic Society <https://www.autism.org.uk/>
- Organization for Autism Research <https://researchautism.org/>
- RU OK? <https://www.ruok.org.au/neurodivergent>
- Samaritans <https://www.samaritans.org/>
- WellChild <https://www.wellchild.org.uk/>

**Supplementary File 4: Data Extracted**

A standardised data extraction coding set will be developed and piloted by the review team on a selection of included studies and refined if needed. It will be used to collect the following information from each included full text:

- First author
- Date of publication
- Title of publication
- Study location
- Study focus
- Study aims and objectives
- Study design
- Data collected
- Data collection method
- Data analysis performed
- Study setting
- Inclusion criteria
- Participant characteristics (age, gender, diagnosis of autism, possible autism)
- Intervention name and aim
- Intervention and comparator characteristics
- Comparator name and aim
- All outcomes assessed in study
- Evidence relating to effectiveness and economic effectiveness
- Evidence relating to experiences of, attitudes and perceptions on, interventions or designed interventions to reduce suicidality
- Summary of key findings (based on paper abstract)

**Supplementary File 5: Stakeholder consultation and its impact on the review process**

| **Stage of review** | **Method of stakeholder involvement** | **Impact on review** |
| --- | --- | --- |
| **Research question & protocol development** | 1x60min meeting via MT with DHSC stakeholders.  Communication via email with DHSC stakeholders  Sharing video-lay summary via email with PERSPEX members  1x20min discussion of review topic via MT with PERSPEX members | Provided context for review and revised review aims and research questions. Through email discussion, we agreed this review would not include studies focusing on assessing the validity or reliability of assessment tools, or exploring epidemiological factors associated with suicide risk. We also agreed this review would include pharmacological interventions and medical procedures, such as electroconvulsive therapy (ECT).  DHSC and NHSE stakeholders reinforced necessity of studies reporting suicide specific outcomes and supported the refining of bibliographic database search terms and key websites to search and refining of inclusion criteria.  Both DHSC and PPI stakeholders helped to identify potential websites to include in search strategy. |
| **Data extraction and quality appraisal** | Communication via email with DHSC stakeholders | Approved content of data extraction form. |
| **Summary of key findings and write up** | Communication via email with government stakeholders  1x30min meeting via MT with members of PERSPEX. Communication via email | Provide feedback on review findings and identification of key messages to inform discussion of revised draft.  PPI informed further research section within the discussion section of this report, particularly the need to consider non-Westernised types of intervention and need to explore gender differences in identification and treatment of suicide risk. PERSPEX members expressed no preference for terminology when referring to autistic people or those who use services (i.e. “patient” vs “service user”. Terms used throughout report are “person” and “autistic people” at request of DHSC stakeholders. |
| **Write up** | Communication via email with government stakeholders | Reviewed and provided feedback on draft report prior to it being finalised |
| **Dissemination** | **PLANNED: Communication via email with government stakeholders**  1x30min meeting via MT with members of PERSPEX. Communication via email | Consultation regarding dissemination material required, including identification of key audiences and communication of key messages arising from the review.  Support identifying findings relevant to autistic persons and other members of the public and developing plain language dissemination materials. |

DHSC=Department of Health and Social Care, MT=Microsoft Teams

**Supplementary File 6: Table 1 Characteristics of Studies**

| **Author, Year [Country]: Type of publication. Number of positive QA items/Total QA items** | **Study focus** | **Title** | **Aim of study** | **Intervention aim**  **[Suicidality as primary or secondary target of intervention]** | **Study design: Participants (N)** | **Summary of key findings^a^** |  |
| --- | --- | --- | --- | --- | --- | --- | --- |
| **Intervention: Safety Planning** | | | | | | | |
| Bal 2024 [USA]: JA  QA: 3/7 | Acceptability, Feasibility | The emotional support plan: Feasibility trials of a brief, telehealth-based mobile intervention to support coping for autistic adults | Assess ESP’s acceptability for coping with distress during pandemic and during  PSE | Emotional regulation at times of high stress, not solely at times of suicidal risk  [Suicidality explicitly measured, both primary and secondary intervention target] | Mixed-methods-Pre-post treatment: Autistic people: Adults (26 consented, 21 completed ESP) | Thirty-six autistic adults participated. Most participants reported using strategies from their ESP; 86%–89% reported positive effect of intervention, 67%–71% would recommend to others. Completion of weekly monitoring and outcome assessments high; ecological momentary assessment variable. |  |
| Goodwin 2024 [UK]: Pre-print JA | Development | Adapting safety plans for autistic adults with involvement from the autism community | Seek advice from autistic adults and others in autism community on how to adapt safety plans for autistic adults | Reduce risk of self-harm/suicide.  [Suicidality primary intervention target] | Qual-Autistic people, family members, service providers (46) | Theme one: conditions needed to make the process of creating AASP acceptable for autistic adults. Included creating AASP with someone they could trust and at right place and time, when they were not in distress or in crisis. Theme two: how safety planning needed to be a creative, flexible, and iterative process. Autistic adults may need help in expressing emotions and identifying coping strategies, which can be supported through visual resources and suggestions from service provider. To ensure AASP is accessible in times of crisis, needs to meet autistic adults’ preferences regarding formatting and how stored. |  |
| NCT 2022 (Kalb 2022) [USA]: TRI | Efficacy, feasibility, utility | A Crisis Prevention Program in Youth with Autism Spectrum Disorder | Examine: 1) Child and parent outcomes associated with novel crisis prevention program for autistic children; 2) Gather survey-based feedback from parents, post-intervention, to understand feasibility and utility of crisis prevention program | Provide families with tools before child's behaviour becomes acute crisis  [Suicidality included as one of a range of potential behaviours which challenge] | Quant-RCT: Parent of child with autism (NR) | Trial completed, not published |  |
| Rodgers 2024 [UK]: JA  QA: 2/7 | Feasibility, Acceptability | Feasibility and acceptability of autism adapted safety plans: an external pilot randomised controlled trial | Primary aim: Establish feasibility and acceptability of AASP and inform parameters of definitive RCT. Secondary aims: explore extent clinical and health economics outcomes can be accurately measured and reported in future definitive trial | Reduce risk of self-harm/suicide.  [Suicidality primary intervention target] | Mixed-methods-Quantitative+Qual- Feasibility RCT: Autistic people (adults) (53) | Feedback on AASP and research methods were positive with suggested adaptations to some outcome measures. Retention and completion of outcomes measures in both arms was excellent, as was fidelity of delivery of AASP |  |
| NCT 2022 (Roubinov 2022) [USA]: TRI | Effectiveness, Feasibility, Acceptability | A Comparison of Two Brief Suicide Prevention Interventions Tailored for Youth on the Autism Spectrum | Compare effectiveness, feasibility, and acceptability of two suicide prevention strategies tailored for autistic individuals: the SPI tailored for Autistic individuals (SPI-A) and SPI-A plus structured follow-up contacts (SPI-A+). Aim 1: Among autistic youth (age 12-24) at risk for suicide, compare effectiveness of SPI-A vs. SPI-A+. Aim 2: Compare implementation outcomes of acceptability and feasibility for two interventions from perspectives of patients, clinicians, and health system administrators. Aim 3: Explore patient characteristics that may moderate relationship between intervention and SI and SB | Reduce risk of self-harm/suicide.  [Suicidality primary intervention target] | Quant-RCT: children/young people, clinicians and Health System Leader Participants (1665) | Trial in progress |  |
| **Intervention: Dialectical Behaviour Therapy** | | | | | | | |
| Bemmouna 2022 [France]: JA  QA: 2/7 | Feasibility, Acceptability and Effectiveness | Feasibility, Acceptability and Preliminary Efficacy of Dialectical Behaviour Therapy for Autistic Adults without ID: A Mixed-methods Study | Assess feasibility, acceptability and preliminary efficacy of DBT in autistic adults without ID exhibiting self-harm and/or suicidal behaviours linked to severe emotional dysregulation | Reduce risk of self-harm/suicide.  [Suicidality primary intervention target] | Mixed-methods-Pre-post treatment, single arm: Autistic people (adults) (7) | DBT feasible and highly acceptable to autistic adults without ID. Difficulties in Emotion Regulation Scale mean scores decreased significantly post-treatment and at 4-month follow-up |  |
| Huntjens et al 2024 [NL]: JA  QA: 6/7 | Effectiveness | The effectiveness and safety of dialectical behaviour therapy for suicidal ideation and behaviour in autistic adults: a pragmatic RCT | Evaluated the effectiveness of DBT vs TAU in reducing suicidal ideation and suicide attempts | Reduce risk of self-harm/suicide.  [Suicidality primary intervention target] | Quant-RCT: Autistic people (123) | At end-of-treatment, DBT significantly reduced suicidal ideation compared to TAU, but lost statistical significance at 12-month follow-up. Depression severity significantly decreased with DBT remaining so at 12 months. No effects on social anxiety. SAE included two suicides in TAU condition |  |
| NCT 2022 (Lobregt-van Buuren 2022) [Netherlands]: TRI | Effectiveness, Experiences | Development of Self-regulation by Dialectical Behavioural Therapy in Adults with Autism | Experiences of adults with ASD and severe emotion dysregulation with Integrative DBT and mechanisms and processes that hinder/ advance recovery to make treatment more tailored and effective for this group. Aim 1: Quantify effectiveness of integrative DBT in adults with ASD and difficult to treat severe emotion-dysregulation and maladaptive coping. Aim 2: Determine improvements over time in sensory hyper- and hyposensitivity, interoceptive body-awareness, cognitive and behavioural emotion-regulation, and well-being+ explore how responders, non-responders and deteriorators differ regarding autistic traits, PTSS-symptoms, sensory hyper- and hyposensitivity and interoceptive body-awareness. Aim 3: Determine how qualitative findings regarding patients' experiences with integrative DBT enhance understanding of quantitative clinical outcomes to get insight in process and sustainability of self-regulation, and make treatment more tailored and effective in recovery | Reduce risk of self-harm/suicide.  [Suicidality one of primary intervention targeted as part of ‘Maladaptive coping: using harmful behaviours’] | Mixed-methods-(i) RCT (ii) quasi-experimental study (iii) qualitative study: Autistic people (adults) (30) | Trial in progress |  |
| NCT 2021 (Weiner 2021) [France]: TRI | Efficacy | Dialectical Behaviour Therapy for Adults with Autism Spectrum Disorders Without ID: a RCT | Assess efficacy of 5-month DBT intervention in adults with ASD without ID who present with self-harm and/or suicidal behaviours | Reduce risk of self-harm/suicide.  [Suicidality one of primary intervention targeted as part of managing emotional dysregulation] | Quant-RCT: Autistic people: adults (NR) | Trial in progress |  |
| Santomauro 2016 [Australia]: JA  QA: 4/7 | Effectiveness, Feasibility, Acceptability | Depression in Adolescents with ASD: A Pilot RCT of a Group Intervention | Evaluate feasibility, acceptability and preliminary efficacy of cognitive behavioural intervention for depression in adolescents with ASD. Research questions: 1) Is it feasible to recruit and engage adolescents with ASD and depression through a CBT programme that aims to address depressive symptomatology? 2) Do adolescents with ASD find programme acceptable and useful? 3) Does CBT programme reduce self-reported symptoms of depression the use of expressive suppression, and increase the use of cognitive reappraisal? 4) Do effects of programme remain stable 3 months post-intervention? 5) Are ToM difficulties associated with self-reported emotion regulation skills and symptoms of depression? | Reduce depression  [Suicidality as a secondary outcome as measured on Beck Depression Inventory. Participants at high risk of suicide were excluded from study.] | Quant-Pilot RCT: Autistic people: adolescents (23) | Recruitment difficult, attendance favourable, attrition low. Participants satisfied with programme. No significant treatment effect on BDI or Emotion Regulation Questionnaire. Despite small sample size (n = 20), trending treatment effect measured by DASS: Depression Subscale |  |
| Shafique 2023 [Pakistan]: Website Abstract | Feasibility, Acceptability | A Pilot Trial of a Culturally Adapted Intervention to Prevent Self-harm in Young Autistic people | Evaluate feasibility and acceptability of adapted Youth Culturally adapted manual Assisted Psychological intervention (YCMAP) based on CBT for young autistic people in Pakistan (evidence-based intervention for reducing self-harm in the general population) | Reduce self-harm and suicidal behaviour  [Suicidality a primary intervention target] | Quant-Pilot RCT: Autistic people (80) | Trial in progress |  |
| **Intervention: Psychosocial therapy-other** | | | | | | | |
| Cashin 2013 [Australia]: JA  QA: 6/7 | Effectiveness, Feasibility | The Effectiveness of Narrative Therapy With Young Autistic people | Determine whether narrative therapy effective in helping young autistic people with emotional and behavioural problems | Reduce stress related problems  [Hopelessness primary intervention target]* | Quant-Pre-post treatment: Autistic people (children) (10) | Signiﬁcant improvement in psychological distress and emotional symptoms. Cortisol:DHEA ratio responsive with power analysis indicating further study with larger sample |  |
| Schiltz 2018 [USA]:  QA: 4/7  JA | Effectiveness | Changes in Depressive Symptoms Among Adolescents with ASD Completing the PEERS® Social Skills Intervention | Examine: 1) Social skills intervention (PEERS®) impacts multiple dimensions of depressive symptoms among adolescents with ASD in USA, 2) Relation between direct peer interactions and depression at intervention post-test, and 3) Changes in self-reported suicidal ideation | Enhance social skills and reduce depression and thereby reduce suicidality.  [Suicidality secondary intervention target] | Quant-RCT: People with ASD (adolescents) (49) | Significant decline in CDI subscale scores, related to direct social contact on the Quality of Socialization Questionnaire at post-test (p’s < 0.05). Suicidality less evident following PEERS® |  |
| **Intervention: Training** | | | | | | | |
| Cervantes 2023a [USA]: JA  QA: 4/7 | Perceptions | Assessing and Managing Suicide Risk in Autistic Youth: Findings from a Clinician Survey in a Paediatric Psychiatric Emergency Setting | 1) Examine differences in knowledge/ attitudes on suicide-related care across autistic patient population and general child and adolescent patient population. 2) Identify factors that improve clinician confidence and quality of care, by examining impact of previous training on ASD topics on clinician confidence+ identifying and managing STB in autistic youth | Effects of training on clinician confidence in identifying and managing suicidal thoughts and behaviours  [Suicidality secondary intervention target | Quant-Cross-sectional: Paediatric psychiatric ED who are mental health specialists and work with autistic patients, but not necessarily specialized in ASD care (16) | While clinicians rated addressing STB in ASD as important and adaptations to care as necessary, half identified ASD as a suicide risk factor and confidence ratings were significantly lower for autistic patients. Previous ASD training predicted confidence and accounted for approx. 25% of variance in confidence scores |  |
| Cervantes 2023b [USA]: Website Abstract | Perceptions, Development | Barriers and Facilitators to Suicide Risk Screening and Management for Autistic Youth | Contribute to comprehensive understanding of barriers and facilitators to implementation of suicide risk screening and management from perspectives of specialized and non-specialized mental health clinicians and caregivers of autistic youth. Goals: 1) Evaluate mental health clinicians’ and parent attitudes/perspectives to understand how they facilitate or reduce screenings and treatment for autistic youth. 2) Develop educational materials to support parents/clinicians in addressing suicide risk with autistic youth and evaluate for acceptability/utility | Education component: Implement suicide risk screening  [Suicidality implicit target of intervention; NR if measured] | Mixed-methods: cross-sectional: specialized and non-specialized mental health clinicians and caregivers of autistic youth (Survey: 125 (Aim) Qualitative interviews: 25) | Trial in progress |  |
| Zoha 2022 [USA]: Abstract( | Perceptions, Effectiveness | 2.100 Identifying Gaps in Provider Knowledge: Trainees Less Comfortable with Suicide Assessment in Youth with ASD and ID | Examine changes in confidence level in assessing and addressing STB before and after SAFETY-A training in an urban public hospital | Promote clinician confidence in assessing for suicide risk in autistic adolescents  [Suicidality implicit target of intervention; NR if measured] | Quant-Cross-sectional: Clinicians (10) | Participants were least comfortable assessing STBs in youth with an ID and ASD. After completing SAFETY-A, trainees’ confidence in treating STBs in children and adolescents with an ID and ASD increased significantly |  |
| **Intervention: ECT/rTMS** | | | | | | | |
| Consoli 2013 [France]: JA  QA: 6/7 | Effectiveness | ECT in adolescents with ID and severe self-injurious behaviour and aggression: a retrospective study | Assess efficacy of ECT on severe and treatment-resistant SIB/AGG in young people with ID and current psychiatric disorder | Reduce SIB  [Suicidality one example of SIB exhibited by one autistic person] | Quant-Retrospective chart review: Children (4) | ECT associated with significant decrease in SIB/AGG (p<0.001): mean aggression scores post-ECT half the pre-ECT value |  |
| NCT 2021 (Ameis 2024) [Canada]: TRI | Efficacy | rTMS for Depression in Young Adults with Autism (rTMS-MDD) | Evaluate efficacy of rTMS for treatment of depression in youth and young adults with ASD | Reduce depressive symptoms  [Suicidality secondary treatment target] | Quant-RCT: Autistic people: youth/young adults (80 (aim)) | Trial in progress |  |
| **Screening: Ask Suicide Questionnaire** | | | | | | | |
| Cervantes 2024c [USA]: JA  QA: 6/7 | Effectiveness | Identification of Suicide Risk in a Paediatric Psychiatric Emergency Setting: Comparing the ASQ and the K-CAT-SS | Compare K-CAT-SS and ASQ, a widely used measure in EDs, in participants presenting to paediatric, psychiatric emergency setting | NA | Quality improvement study: quantitative, cross-sectional: young people and carers (329: 183 youth and 146 caregivers (146 dyad screens, 37 youth only screens) included in analyses) | Measures agreed on presence of suicide risk in >85% of cases (κ = 0.59), and characteristics of youth who screened at risk on both were similar. Cases of disagreement more often male, had educational accommodations, lower symptom levels and less often diagnosed with internalizing disorders and were less often identified as high risk by ED psychiatrists and psychologists. Examination of item endorsement patterns in cases of disagreement revealed important areas of future study, including the role of caregiver report in suicide risk screening, item comprehension concerns, and validity of assessing youth with neurodevelopmental disabilities. While additional research would be beneficial into its psychometrics when deployed in real-world settings, K-CAT-SS a viable alternative for suicide risk screening in EDs |  |
| Horowitz 2018 [UK]: Abstract | Feasibility | 3.2 Suicide Risk in Youth with ASD: Feasibility of Screening in the Medical Setting | 1) Review existing data on suicide risk in youth with ASD. 2) Report preliminary feasibility data from several studies adapting/testing the ASQ toolkit for populations with ASD | NA | Quant-Cross-sectional: people with ASD, parents (14 People who use services, 4 parents) | Four patients (28.6%) screened positive for suicide risk on SIQ-CV. Among these 4 only one had documented history of both suicidal thoughts and/or behaviours |  |
| Kalari 2020 [USA]: Abstract | Effectiveness | 3.31 Now Screening-Suicidality and ASD: Can the ED Play a leading role? | Evaluates utility of universal suicide screening using the ASQ in children and adolescents with ASD who presented to large urban PED | NA | Quant-Retrospective chart review: people with ASD (children) (18,357 patients - 393 with ASD) | When combining all types of visits, 12.5% of those with ASD and 3.8% of those with no ASD reported suicidality as chief concern. Among children who did not report suicidality as chief concern, more from ASD group screened positive on ASQ than from no-ASD group (22.9% vs 12.1%). Item-level data showed 21.1% of children with ASD had thoughts of attempting suicide in the past week compared to 7.4% of those without ASD. 16.5% of those with ASD reported a past suicide attempt compared to 7.3% of youth without ASD |  |
| Rybczynski 2022 [USA]: JA  QA: 4/7 | Implementation | Suicide Risk Screening in Paediatric Outpatient Neurodevelopmental Disabilities Clinics | Describe implementation of universal suicide risk screening in paediatric NDD medical clinics, analyse demographic and clinical characteristics of eligible patients, describe outcomes of positive screenings, and describe factors influencing screening participation | NA | Quant-Retrospective chart review: People who use services, carers (Autism and related disorder: 699 (70.2% eligible in individual clinic, 18.14% of total people screened). 84 (12%) screened positive)) | During 6-month study period, 2961 individual patients presented for 5260 screening eligible patient visits. 3854 (73.3%) completed screenings with 261(6.8%) positive screenings noted. Screenings were declined in 1406 (26.7%) visits. Parents of children with cognitive impairments were more likely to decline screening. Clinics serving children with ASD had higher rates of positive screenings compared with other clinic attendees. Seventy-two of 187 children (38.5%) with positive screenings were identified and referred to outpatient mental health referrals. Seven (2.5%) of these children required acute psychiatric treatment |  |
| Vasa 2017 [USA]: Abstract( | Effectiveness | 3.55 Assessment of Suicide Risk in Children and Adolescents with Autism Spectrum Disorder Presenting to a Paediatric Emergency Department | Compare efficacy of eliciting chief concern upon presenting to ED vs ASQ, administered at triage, in detecting suicidality in children and adolescents with ASD in the ED setting. Examine characteristics of children/adolescents with ASD who reported suicidal ideation in ED | NA | Quant-Retrospective chart review: children and adolescents with ASD (104) | Among the 104 participants with ASD, 31 screened positive on ASQ for suicide risk. Sixty-five percent of patients with ASD uniquely identified as experiencing suicidal ideation by the ASQ. Suicide attempts reported among 12 youth and consisted of following methods: stabbing/cutting (n=5), jumping from a height (n=2), choking/holding breath/hanging (n=3), overdose (n=1), and firearms (n=1) |  |
| **Screening: Universal Tool in autistic population** | | | | | | | |
| Cassidy 2020 [UK]: JA  QA: 7/7 | Usability | Measurement Properties of the Suicidal Behaviour Questionnaire Revised in Autistic Adults | Explore appropriateness and measurement properties of widely used and validated suicidality assessment tool developed for general population, in autistic adults. Will inform adaptations to better capture suicidal thoughts/ behaviours in autistic adults. Research questions (i) Does suicidality assessment tool (SBQ-R) validated in general population similarly capture latent construct of suicidality in autistic adults? (ii) Do autistic adults interpret and respond to SBQ-R questions as intended, and if not, how can tool be adapted to better capture suicidality in this group? Focus is on appropriateness | NA | Sequential, explanatory mixed-methods design (Qualitative component only): Autistic adults (15-a subgroup of those who completed online survey) | 188 autistic adults+183 general population adults completed tool. Sub-sample (n=15) interviewed while completing tool. Cognitive interviews revealed autistic adults did not interpret items as intended by tool designers and interpret key questions regarding suicide risk differently to general population |  |
| Cervantes 2024a [USA]: JA  QA: 6/7 | Usability | Perspectives from the Autism Community on the Potential Utility of a Novel Measure of Suicide Risk and Mental Health Symptoms for Autistic Youth: A Pilot Study | Obtained feedback from autistic youth, caregivers, and autism specialist clinicians (N = 14) on the applicability of a novel measure of suicide and mental health symptoms, the Kiddie-Computerized Adaptive Test (K-CAT) scales, for use with autistic youth. The study aimed to develop emergency department (ED) clinician training in autism and suicide and to evaluate a screening program for autistic youth implemented within a larger initiative introducing the K-CAT to improve suicide risk screening in EDs for all youth aged 7–17 years old. The goals of the study were to both evaluate general impressions of the full K-CAT and identify specific concerns with the K-CAT-SS to begin to understand the utility of the measure for autistic youth | NA | Mixed-methods pilot study: questionnaire and qualitative interviews: young people with autism, caregivers, autism specialist clinicians (14) | Impressions were largely positive and several features support its use but participants identified several concerns. These aligned with those identified in previous research on other measures developed for the nonautistic population, with the most endorsed problem being language/terminology issues. Additional areas of assessment (e.g., perseveration, emotion dysregulation) were recommended to better capture the experience of suicidality in autistic youth. Results continue to underscore that measures developed for the general population cannot be applied to autistic individuals without thorough evaluation and potential modification. This feedback will be used in a future modification of the K-CAT for use with autistic youth |  |
| Kalb et al., 2022 [USA]: JA  QA: 5/7 | Feasibility | Mental Health Crisis Screening in Youth with Autism Spectrum Disorder | Examining 1) Feasibility and utility of conducting routine crisis screenings. 2) Psychometrics of brief crisis screener MCAS-R. c) Prevalence of and types of behaviours associated with crises. Aim 1: Determine feasibility of implementing MCAS as a screening tool in outpatient clinic setting, determined by proportion of sample successfully screened. Aim 2: Examine psychometric properties of revised version of MCAS, which was based on findings from this study including how well it aligned with clinician determination of crises in outpatient clinics. Aim 3: evaluate prevalence of mental health crises in outpatient clinics and types of behaviours that contributed to the crisis. Aim 4: examine parent and clinician satisfaction with crisis screening protocol | NA | Quant-Cross sectional (service improvement project): Caregivers of youth with autism (406 children screened (Respondents on behalf of children: 332 Mothers, 57 Fathers, 15 "Other") | 80% youth successfully screened, suggesting crisis screening is feasible. Most parents (73%) felt MCAS-R helped communicate concerns with clinician; <6% felt survey was too long or upsetting. All clinicians indicated that MCAS-R was very helpful in facilitating communication and identifying/mitigating safety concerns; although 33% reported screenings “sometimes” interrupted clinical flow. MCAS-R strongly aligned with clinician ratings (88% correctly classified). 20% youth met cutoff for crisis; aggression and self-injurious behaviours were most common reasons for crises |  |
| **Screening: Experiences** | | | | | | | |
| Cervantes 2024b [USA]: JA  QA: 7/7 | Develop guidelines, Experiences | Improving Emergency Department Care for Suicidality in Autism: Perspectives from Autistic Youth, Caregivers, and Clinicians | Develop recommendations for modifying ED care for autistic patients, focusing on suicide risk screening and management | NA | Pilot study: Qualitative interviews: Young people, carers and autism specialist clinicians (17 participants (5 young people/caregiver dyads, 1 caregiver without young person, 3 Autism Specialist Clinicians, 3 ED clinicians) | Participants reported on challenges encountered receiving or providing care and/or recommendations for improving care. Participant perspectives were aligned. Four main categories: accounting for autism features, connection and youth engagement in care, caregiver and family involvement, and service system issues |  |
| Jager-Hyman 2002 [USA]: JA  QA: 5/7 | Intervention/ Screening: Perceptions | Mental Health Clinicians’ Screening and Intervention Practices to Reduce Suicide Risk in Autistic Adolescents and Adults | 1) Examine clinicians’ use of and beliefs about suicide risk screening practices for autistic adolescents and adults in comparison to adolescents and adults without autism. 2) Examine clinicians’ knowledge of and confidence in using SPI with autistic clients relative to clients without autism | NA | Quant-Cross sectional: Clinicians (121 started survey) | Clinicians reported greater self-efficacy screening for suicide risk among non-autistic clients (p=0.01). No statistically sig. differences in whether they used standardized screening measures or in their reported normative pressure or attitudes towards screening. Clinicians reported similar rates of use of Safety Planning, an evidence-based suicide-prevention strategy, across groups, but significantly greater acceptability for non-autistic clients |  |

*High correlation suicidality. Blue shaded cell=articles from same study. ^a^based on article abstract, Appropriateness=clarity and relevance of items in a tool to the group(s) in which the tool is intended to be used. AASP=Autism Adapted Safety Plan, AGG=Aggression, ASD=Autistic Spectrum Disorder, ASQ= Ask Suicide-Screening Questions, BDI=Beck Depression Inventory, CBT=Cognitive Behavioural Therapy, CDI=Children's Depression Inventory, DASS=Depression Anxiety Stress Scale, DBT=Dialectical Behaviour Therapy, DHEA=Dehydroepiandrosterone, ECT=Electroconvulsive Therapy, ED=Emergency Department, ESP=Emotional Support Plan, ID=Intellectual Disabilities, JA=Journal Article, K-CAT-SS=Kiddie-Computerized Adaptive Test-Suicide Scale, MCAS-R=Mental Health Crisis Assessment Scale Revised;, N=Number, NDD=Neurodevelopmental Disabilities, NR=Not Reported, PED=Paediatric Emergency Department, PSE=Post-secondary Education, PTSS=Post-Traumatic Stress Symptoms, QA=Quality Appraisal, SBQ-R=The Suicide Behaviours Questionnaire-Revised, RCT=Randomized Controlled Trial, rTMS=Repetitive Transcranial Magnetic Stimulation, SAE=Severe Adverse Events, SIB=Self-Injurious Behaviour, SIQ-CV= Suicidal Ideation Questionnaire-Child Version, SPI=Safety Planning Intervention, STB=Suicidal Thoughts and Behaviours, TAU=Treatment as Usual, TRI=Trial Registry, UK=United Kingdom, USA=United States of America

**Supplementary File 7: Critical appraisal of included studies**

**Table 1** MMAT quality appraisal - qualitative studies

|  | **SCREENING QUESTIONS** | | **1. QUALITATIVE STUDIES** | | | | | **COMMENTS** |
| --- | --- | --- | --- | --- | --- | --- | --- | --- |
| **First author, year** | Are there clear research questions? | Do collected data allow to address research questions? | Is qualitative approach appropriate to answer research question? | Are qualitative data collection methods adequate to address research question? | Are findings adequately derived from data? | Is interpretation of results sufficiently substantiated by data? | Is there coherence between qualitative data sources, collection, analysis and interpretation? |  |
| Cassidy et al 2020 | Y | Y | Y | Y | Y | Y | Y | Good PPIE work done in preparation for cognitive interviews (pre-prepared prompts) |
| Cervantes 2024a | Y | Y | Y | Y | Y | N | Y | Lack of supporting participant quotes to evidence results identified |
| Cervantes 2024b | Y | Y | Y | Y | Y | Y | Y | Pilot study: small sample size for each category of participant |
| Goodwin 2024 | Y | Y | Y | Y | Y | Y | Y | NA |

**Table 2** MMAT quality appraisal – RCTs

|  | | **SCREENING QUESTIONS** | | | | **2. RANDOMIZED CONTROLLED TRIALS** | | | | | | | | | | **COMMENTS** | |
| --- | --- | --- | --- | --- | --- | --- | --- | --- | --- | --- | --- | --- | --- | --- | --- | --- | --- |
| **First author, year** | | Are there clear research questions? | | Do collected data allow to address research questions? | | Is randomization appropriately performed? | | | Are groups comparable at baseline? | | Are there complete outcome data? | | Are outcome assessors blinded to the intervention provided? | | Did participants adhere to assigned intervention? |  |  |
| Huntjens et al 2024 | | Y | | Y | | Y | | | Y | | Y | | Y | | N | Pre-planned sensitivity analysis conducted for main analyses (SIDAS suicidal ideation, LPC suicide attempts, BDI depressive severity, SIAS social anxiety) in which missing observations due to dropout imputed using LOCF | |
| Santomauro 2016 | | Y | | Y | | CT | | | Y | | Y | | CT | | N | Whilst scores on AQ at baseline differed significantly between groups, was accounted for in analyses. Participants reported poor homework compliance | |
| Schiltz 2018 | | Y | | Y | | CT | | | Y | | Y | | CT | | N | Ratings based on content of this paper. Checked against content reported in Schohl 14([53](#_ENREF_53)) | |
| AQ=Autism Spectrum Quotient, BDI=Beck Depression Inventory, CT=Can't Tell, LOCF=Last-Observation-Carried Forward. LPC=Lifetime Parasuicide Count, N=No, SIDAS=Suicidal Ideation Attributes Scale, Y=Yes  **Table 3** MMAT quality appraisal - quantitative descriptive studies | | | | | | | | | | | | | | | | | |
|  | | **SCREENING QUESTIONS** | | | | **4. QUANTITATIVE DESCRIPTIVE STUDIES** | | | | | | | | | **COMMENTS** | | |
| **First author, year** | | Are there clear research questions? | | Do the collected data allow to address the research questions? | | Is sampling strategy relevant to address research question? | | Is sample representative of target population? | Are measurements appropriate? | | Is risk of nonresponse bias low? | | Is statistical analysis appropriate to answer research question? | |  |  |  |
| Cashin 2013 | | Y | | Y | | Y | | N | Y | | Y | | Y | | Designed as small single-sample, unblinded pilot study to evaluate effectiveness of narrative therapy across a range of psychological and biological measures. No. participants (10, all male) too low to establish if truly representative of sample population | | |
| Cervantes 2023a | | Y | | Y | | CT | | CT | Y | | CT | | Y | | Sample size small, consisting of primarily young, female clinicians | | |
| Cervantes 2024c | | Y | | Y | | Y | | Y | Y | | N | | Y | | 73 dyads of 334 eligible declined to participate | | |
| Consoli 2013 | | Y | | Y | | Y | | N | Y | | Y | | Y | | Sample not representative due to limited size | | |
| Jager Hyman 2020 | | Y | | Y | | Y | | CT | Y | | N | | Y | | NA | | |
| Kalb et al 2022 | | Y | | Y | | Y | | CT | Y | | N | | Y | | Only 61% of eligible participants screened at one site (80% overall response rate across both sites). Differences between responder’s vs non-responders not explored | | |
| Rybczynski 2022 | | Y | | Y | | Y | | CT | Y | | N | | CT | | NA | | |
| CT=Can't Tell, N=No, NA=Not Applicable, Y=Yes | | | | | | | | | | | | | | | | | |

**Table 4** MMAT quality appraisal - mixed methods studies

| **First author, year** | **SCREENING QUESTIONS** | | **MIXED METHODS STUDIES** | | | | | **COMMENTS** |
| --- | --- | --- | --- | --- | --- | --- | --- | --- |
|  | Are there clear research questions? | Do the collected data allow to address the research questions? | Is there an adequate rationale for using a mixed methods design to address the research question? | Are the different components of the study effectively integrated to answer the research question? | Are the outputs of the integration of qualitative and quantitative components adequately interpreted? | Are divergences/ inconsistencies between quantitative and qualitative results adequately addressed? | Do different components of study adhere to quality criteria of each tradition of methods involved? |  |
| Bal 2024 | Y | Y | N | Y | N | N | N | Qualitative analysis very "descriptive". Minimal integration with results from structured interviews. Could be considered appropriate as data complimentary and "quant" |
| Bemmouna 2022 | Y | Y | N | N | N | N | N | Methods pertaining to qualitative data rationale, collection and analysis poorly reported. Results of quant and/qual also poorly integrated. Uncertain that the confidence with which conclusions reported is justified given methodological limitations acknowledged by the authors re: study design, participant characteristics and limited sample size |
| Rodgers 2024 | Y | Y | N | N | N | N | N | Conduct, analysis and results of qualitative interviews inadequately reported |

CT=Can't Tell, N=No, Y=Yes

**Supplementary File 8: Studies evaluating interventions**

| **Intervention** | **Description** | **Participants** | **Key features** | **Delivery, dose, setting** | **Comparator** | **Outcomes evaluated** |
| --- | --- | --- | --- | --- | --- | --- |
| **Interventions: Safety Planning** | | | | | | |
| Emotional Support Plan  (Bal, 2023) | **Aim:** Support autistic adults to use positive coping skills during periods of distress  The **emotional support plan** (ESP) has six main sections:  (i) Warning signs: helps individuals identify signs that they are beginning to feel stressed or upset  (ii) Things I can do to manage my emotions: help the participant identify coping strategies  (iii) Positive supports: people that can serve as contacts to provide support  (iv) Reasons for controlling my emotions: consider possible consequences or outcomes associated with losing control due to feelings of stress  (v) Ways that I can make my environment less stressful: identify changes to the environment to reduce stress  (vi) Professional or other help: identify professional sources of support and the associated contact information  Two final sections, drawn from the Stanley–Brown Safety Plan, are optional for individuals with a history of suicide risk to promote safety during those specific circumstances.  (vii) Reasons for living: individuals to think about reasons to live and things that are important to them  (viii) Ways that I will make my environment safer: identify and remove the individual’s access to lethal or dangerous items | **Who**  People who use services (adults)  **Number**  26 consented, 21 completed  **Age**  COVID-C group: 25.1(7.1), PSE-C group: 19.5(6.5), PSE-S group: 21.5(3.1)  **Gender**  Women: 13 (36%)  **Autism diagnosis**  Y | **Adapted for autism:**  both clinician- and self-guided versions of the ESP were designed and feasibility-tested in recognition that many autistic adults are seeking interventions that could be self-initiated or managed. Two autistic graduate students were members of the study team and they participated in co-development of the ESP-S (self-guided) videos and other materials and with the monitoring visits for the PSE study.  **Theory informed:**  Mirrors the structure of the Stanley–Brown Safety Plan (Stanley & Brown, 2012) | **Who delivers**  Clinician and self-guided  **How**  Telehealth and mobile  **For how long**  15 weeks  **Where**  NR  **Completed**  Y | NAF | Acceptability: Patient satisfaction  Feasibility: Recruitment rate  Feasibility: Screening rate  Feasibility: Withdrawal rate  Feasibility: Outcome completion  Acceptability: Weekly stress ratings |
| Safety Planning  (Goodwin 2024; Rogers 2024) | **Aim**: Reduce self-harm and suicide for autistic people Adapted Safety Plans (AASP)  The **original Stanley & Brown safety plan** consists of six sections involving identification of:  (i) warning signs  (ii) internal coping strategies (iii) social contacts and locations  (iv) family members or friends that may offer help  (v) professionals or agencies to help  (vi) how to keep the environment safe.  The AASP adapted Stanley & Brown’s safety plans in partnership with autistic adults and those who support them. The AASP was accompanied by an optional resource pack, which included tools to identify emotions, scales, pictorial representations, and support services to support the autistic person. This resource pack was developed in collaboration with autistic people, their families, and those who support them through PPI focus groups in stage one, and feasibility interviews in stage two of the study | **Who**  People who use services (autistic adults)  **Number**  53  **Age**  39 (13) [18-70]  **Gender**  Women: 49 (49%)  **Autism diagnosis**  Y | **Adapted for autism:**  Stage 1 PPI adaptations involved clarifying the template to meet autistic thinking and communication styles. Training for support workers and researchers was codesigned with autistic people and included information  about suicide and self-harm in autistic people, adaptations from standard safety planning, considerations when  working with autistic people, helpful insight into autism, such as the double empathy problem (where both autistic and non-autistic people struggle to understand and empathise with one another), opportunities to discuss and practise the AASP.  **Theory informed:**  Suicide safety plans are a series of hierarchical steps to be followed to help people to stay safe during periods of acute crisis that have demonstrated effectiveness in reducing self-harm and suicidal behaviour amongst non-autistic people. They can be delivered by a range of professionals and can be adapted to meet the heterogeneous presentations of autistic people, including sensory and communication preferences or areas of passionate interest | **Who delivers**  One-to-one via support worker or researcher  **How**  Safety plan was completed via telephone or video  **For how long**  NA  **Where**  NA  **Completed**  Y | Treatment as usual (TAU) without AASP  Usual care only, on a one-to-one basis. All participants completed a wellbeing plan with a researcher prior to being randomised. This included information about a trusted person to contact if the research team were worried about a participant's wellbeing. | Acceptability: Completion of AASP  Acceptability: Response rate for completion of outcome measures  Acceptability: Follow up rates  Acceptability: Response rate for questionnaires  Acceptability: Study methods  Acceptability: Data from participants and service providers about what comprises treatment as usual  Acceptability: Percentage of participants who rate the usability of the Safety Plans (SPs) on the System Usability Scale as 68 or above  Satisfaction of people who use services: Percentage of participants who report satisfaction with the AASP intervention  Clinician fidelity: fidelity of delivery of the AASP manual using a  bespoke fidelity checklist  Effectiveness: Self-injurious Thoughts and Behaviours Inventory (SITBI)  Effectiveness: Suicidal Behaviours Questionnaire – Autism Spectrum Condition  Effectiveness: Vulnerabilities Experience Quotient (VEQ)  Economic: healthcare resource utilisation  QoL: Time and travel related to healthcare  QoL: EQ-5D-5L  Satisfaction: System Usability Scale (SUS)  Satisfaction: Client Satisfaction Questionnaire-8 (CSQ-8)19 (administered in the AASP + arm only) |
| Safety planning  (Jager-Hyman 2020) | **Aim:** Reduce the short-term risk of suicide  The clinician collaborates with the autistic person to create a stepwise plan that contains an easy-to-read list of individually tailored, concrete coping mechanisms to be enacted during or leading up to a crisis. The Safety Plan is designed to be clear, concise (one page), and easy to use. The stepwise nature of the plan provides a roadmap for the user; if one step is not effective in reducing suicidal thoughts or urges, the user will then move on to the next step. The coping strategies are initially “within self” (i.e., do not require other people to implement) to encourage self-efficacy. As the steps progress, reliance on external forces for intervention increases, culminating in seeking emergency care | **Who**  Clinicians  **Number**  121 started  **Age**  **Gender**  **Autism diagnosis**  NA | **Adapted for autism:**  **Theory informed:**  The Safety Planning Intervention (SPI; Stanley and  Brown 2012) is one evidence-based suicide prevention  intervention with the potential to be a good fit for autistic  individuals who experience suicidal thoughts or behaviours | **Who delivers**  **NA**  **How**  **For how long**  NA  **Where**  Clinicians worked in community health settings (51%), autism specific clinics (20%), hospital or residential settings (10%), school-based settings (8%), a developmental and paediatric clinic (1%), and other settings (10%), which included outpatient mental health private practices | NA | Knowledge: SPI  Training: use of SPI  Use with autistic and non-autistic people  Acceptability with autistic and non-autistic people |
| Safety Planning  NCT 2022 (Kalb 2022) | **Aim:** Provide families with tools before their child's behaviour becomes acute  The sessions of the **crisis prevention programme** involve case formulation, provision of prevention strategies, connecting with professional and lay resources, what to do if a crisis occurs, and strategies to prevent the re-occurrence of a crisis, should one occur. Each session involves use of checklists, a community resource guide, and homework. Implementation will be standardised using two different manuals, one for the parent and the other for the provider | **Who**  Parents of a child with autism  **Number**  61  **Age**  child/young person (3-17 years)  **Gender**  NR  **Autism diagnosis**  Y | **Adapted for autism:**  NR  **Theory informed:**  NR | **Who delivers**  Licensed clinical therapist  **How**  Via telehealth  **For how long**  Three sessions 60 minutes each  **Where**  NA  **Completed**  **Y** | Placebo  Families in the control condition will receive the Autism Speaks Challenging Behavior Toolkit. It offers a complimentary set of recommendations. There is no interaction with the clinician in the control condition. After the study observation period, we will provide them with the crisis manual (for parents) used in the active treatment arm | Feasibility: Screening response rate  Internal validity: Mental Health Crisis Assessment Scale-Revised (MCAS-R) psychometrics  Parent satisfaction  External validity: Alignment with clinical crisis ratings  Crisis identification  Clinician satisfaction |
| Safety Planning  NCT 2022 (Roubinov, 2022) | **Aim:** Lower short-term risk of suicide in autistic youth  The **Behavioral: Safety Planning Intervention tailored for Autistic Individuals** (SPI-A) involves clinicians working collaboratively with patients, and their family members, to create a list of concrete coping mechanisms to be enacted leading up to or during a crisis. This list can be in writing but can be depicted pictorially, depending on patient preference. As part of the intervention, patients (and family members, when appropriate) also identify warning signs that signal the need to use the safety plan, as well as a detailed plan for reducing access to lethal means. SPI-A is a stand-alone intervention without a follow-up component | **Who**  Children/young people, clinicians and Health System Leader Participants  **Number**  1665  **Age**  Children/young people (12-24 yrs)  Clinicians and Health System Leaders >18 years  **Gender**  NR  **Autism diagnosis**  Y (not explicit) | **Adapted for autism:**  NR  **Theory informed:**  NR | **Who delivers**  Clinicians and Health System Leaders  **How**  NR  **For how long**  **NR**  **Where**  NR  **Completed**  Trial in progress | Comparator: Safety Planning Intervention Tailored for Autistic Individuals Plus Structured Follow-Up Contacts (SPI-A+).  It is a multi-component intervention comprising SPI-A and structured follow-up consisting of at least two brief contacts. The structured follow-up component includes three elements:  (i) brief risk assessment and mood check (ii) review and, if needed, revision of SPI-A (iii) support related to outpatient mental health treatment initiation | Suicidal ideation and suicidal behaviour  Use of mental health services  QoL  Wellbeing  Skills to manage suicidal ideation  Access to lethal means |
| **Interventions: Dialectical Behaviour Therapy (DBT)** | | | | | | |
| Dialectical Intervention Therapy (DBT)  (Bemmouna et al., 2022) | **Aim:** Decrease self-reported emotion dysregulation (ED), improve self-reported depression, hopelessness, alexithymia, perceived quality of life and the frequency and intensity of self-harm and suicidal ideation  **Four components:**  (i) 2hrx15 weekly skills training group sessions  (ii) weekly 1hr individual therapy sessions  (iii) access to telephone coaching  (iv) weekly 2hr therapist consultation  **Four modules of DBT skills:** mindfulness, emotion regulation, distress tolerance and interpersonal effectiveness were covered during 16 skills training sessions. Two debriefing sessions at mid- and end-of-therapy to review skills learned, role play or model skills, answer questions, explain skills, and gather participant feedback | **Who**  People who use services  **Number**  7  **Age**  27.71 (13.34)  **Gender**  Women: 3 (43%)  **Autism diagnosis**  Y | **Adapted for autism: T**herapy was adjusted to the needs of autistic adults: (i) therapy environment kept as stable as possible, individual sessions were on a regular schedule; (ii) Adolescent/Adult Sensory profile pre-treatment skills training (quiet room of clinic); (iii) Pre-treatment psycho-education/problem solving to identify and address barriers to participation in skills-training groups; (iv) activities to normalise anxiety/emotions in first session; (v) modifications to DBT patient manual; (vi) facilitators’ instructions and mindfulness based practices based on concrete examples, role-playing/modelling; (vii) support planning between-sessions practices provided when needed; (viii) participants’ focused interests used in examples during group sessions and incorporated into between-session practices.  **Theory informed:**  Third-wave CBT combining  acceptance-based techniques, including mindfulness and CBT strategies (problem solving, behavioural analysis, contingency management, and skills training) | **Who delivers**  Senior clinical psychologist & a graduate-level clinical psychology intern with training in CBT & DBT  **How**  Group and face-to-face but also individual and virtual because of CoVId pandemic  **For how long**  18 weeks  **Where**  Outpatient psychiatry department (University Hospital of Strasbourg)  **Completed**  Y | NA | Satisfaction of people who use services  Efficacy: emotional regulation  Efficacy: depression  Efficacy: Hopelessness/Depression  Efficacy: Alexithymia  Efficacy: QoL  Attrition  Attendance  Self-harm behaviour  Suicidal thoughts/behaviour |
| Dialectical Intervention Therapy (DBT)  (Huntjens et al., 2024) | **Aim: R**educe suicidal ideation and suicide attempts during the first six months after baseline and to reduce depressive symptom severity and symptoms of social anxiety.  **Four components:**  (i) Weekly 45-minute individual cognitive-behavioural psychotherapy sessions with the primary therapist (these primarily focused on motivational issues, including the motivation to stay alive and remain in treatment)  (ii) weekly 2hr15 skills training group  (iii) if needed, access to telephone coaching with their individual therapist  (iv) weekly 1-hour therapist consultation  **Four modules of DBT skills:** Mindfulness, emotion regulation, distress tolerance, and interpersonal effectiveness skills were covered in the initial 13 weeks and revisited in the program’s second half. 26-week skill training was adapted from Neacsiu et al. (2014) emotion regulation skill training | **Who**  People who use services  **Number**  123  DBT (n = 63) TAU (n = 60)  **Age**  37.4 (11.3)  **Gender**  Women: 58 (47%)  **Autism diagnosis**  Y | **Adapted for autism: T**ext modifications were made to the DBT manual before the study began, simplifying explanations of certain DBT skills to make them more concrete and understandable; mindfulness exercises proposed at the beginning of the skills training were also based on precise and unambiguous instructions tailored to the needs of autistic people. Participants received two pretreatment sessions with their assigned primary DBT therapist, to prepare them for the demands and expectations of DBT, familiarising them with the therapy’s structure, components, and goals.  **Theory informed:**  Combines acceptance-based strategies (e.g. mindfulness and validation) with change-oriented strategies based on second-generation CBT, e.g., problem-solving, behaviour analysis, contingency management, and skills training (Linehan, 1993) | **Who delivers**  Therapist  **How**  Group and individual but shifted to telehealth because of CoVid pandemic  **For how long**  26 weeks  **Where**  Outpatient settings across six Dutch mental health care services  **Completed**  Y | Treatment as usual (TAU). TAU involved at least weekly 45-min sessions with a psychotherapist or social worker. TAU encompassed any common form of treatment for suicidal behaviour in autism within the Dutch mental health system (e.g. emotion regulation therapy, trauma therapy) | Suicidal ideation (primary outcome)  Self-harm  Depression  Social anxiety  Acceptability  Fidelity  Adverse events |
| Dialectical Intervention Therapy (DBT)  NCT 2021 (Weiner 2021) | **Aim:** Reduce emotional dysregulation in adults with ASD (without intellectual disability) who present with self-harm and/or suicidal behaviours.  Psychotherapy will begin within a maximum of one month (the time to set up the group) and will be accompanied by individual follow-up. Evaluations will be carried out within six months of the end of the psychotherapy to measure its effects | **Who**  People who use services  **Number**  NR  **Age**  Adults >18 years  **Gender**  NR  **Autism diagnosis**  Y | **Adapted for autism:**  NR  **Theory informed:**  DBT (Linehan, 1993) effectively diminishes emotion dysregulation and self-harm behaviours in several disorders. | **Who delivers**  NR  **How**  Group and individual  **For how long**  Five months  **Where**  NR  **Completed**  Trial in progress | Wait list.  Wait list for five months during which time the participants will continue their usual therapeutic treatments. Assessments will be carried out within six months after the end of the psychotherapy to measure its effects Participants will benefit from DBT regardless of the group | Efficacy: Emotion dysregulation |
| Dialectical Intervention Therapy (DBT)  NCT 2022 (Lobregt-van Buuren 2022) | **Aim:** Treat severe emotion-dysregulation and maladaptive coping, augmented with a body-oriented DBT-skills training for possible impairments of interoceptive body-awareness.  There will be staggered baselines of four, six, or eight weeks and three patient groups. The treatment at baseline comprises eight weeks outpatient pretreatment DBT, 40 weeks inpatient DBT, 24 weeks follow-up including 12 weeks after care DBT (maximum eight sessions individual DBT and four booster sessions) and 12 weeks no DBT | **Who**  People who use services  **Number**  ~30  **Age**  Adults >18 years  **Gender**  All  **Autism diagnosis**  Y | **Adapted for autism:**  Standard DBT to be adapted to adults with ASD. The inpatient treatment takes place in a living environment that is based on the principles of DBT and considers several aspects of autism.  **Theory informed:**  DBT is an empirically supported psychotherapy to treat severe emotion-dysregulation | **Who delivers**  Trained DBT-therapists and counsellors  **How**  Group and individual  **For how long**  **72 weeks**  **Where**  Inpatient and outpatient  **Completed**  Trial in progress | TAU/Baseline.  Groups 2 and 3 experience six and eight weeks at baseline respectively, before receiving intervention. | Use of harmful behaviours  Self-harm  Suicidal ideation  Interoceptive body-awareness  Emotion dysregulation  Behavioural emotion-regulation  Sensory sensitivity |
| **Interventions: Cognitive Behavioural Therapy** | | | | | | |
| Cognitive Behavioural Therapy  (Santomauro 2016) | Aim: Reduce self-reported symptoms of depression.  ‘**Exploring Depression’ intervention** was designed by Attwood and Garnett (2013) and comprised sessions which explored different ‘tools’ or strategies the adolescents could use in order to manage symptoms of depression:  (i) self-awareness tools  (ii) physical tools  (iii) pleasure tools  (iv) thinking tools  (v) social tools  (vi) relaxation tools.  Each strategy was represented by a hardware tool and the programme represented as a toolbox. These tools were discussed with the adolescents in the sessions, and they were able to choose which tools worked best for them.  Every session assigned home projects for the adolescents to complete before the following session. These involved scheduling the tools they had learned that session into their weekly planner, and completing a self-monitoring sheet to record when they used the tools they had learned, and how they felt before and after they used those tools | **Who**  People who use services (adolescents)  **Number**  23  **Age**  15.75 (1.37)  **Gender**  Women: 11/23 (47.8%)  **Autism diagnosis**  Y | **Adapted for autism:** the first home project was introduced in session two and was to read an article describing the positive qualities of ASD.  **Theory informed:**  The evidence linking emotion regulation skills to depression (Campbell-Sills et al. 2006; Ehring et al. 2011) suggests that interventions focusing on increasing the use of cognitive reappraisal may benefit depressed adolescents with ASD. | **Who delivers**  Two provisionally registered clinical psychologists, supervised by two senior clinical psychologists.  **How**  Group (three to four in group)  **For how long**  10 weeks, booster session in week 14 to recap and gather feedback  **Where**  School of Psychology Clinic at University of Queensland  **Completed**  Y | Wait-list control group | Depression  Depression and anxiety  Emotional/ behavioural problems  Feasibility  Acceptability |
| Cognitive Behavioural Therapy  (Shafique 2023) | **Aim:** Reduce self-harm.  The intervention is a youth culturally adapted manual assisted psychological intervention (YCMAP) based on cognitive behaviour therapy. | **Who**  People who use services (young people)  **Number**  80  **Age**  [18-24]  **Gender**  NR  **Autism diagnosis**  NR | **Adapted for autism:**  NR  **Theory informed:**  NR | **Who delivers**  NR  **How**  Individual  **For how long**  Eight to 10 sessions lasting for 60 minutes.  **Where**  NR  **Completed**  Trial in progress | TAU | Self-harm  QoL |
| **Interventions: Intervention: Psychosocial therapy and narrative therapy** | | | | | | |
| Narrative therapy  (Cashin 2013) | **Aim:** Reduce stress-related problems in young autistic people  **Five sessions:**  (i) know the person away from the problem, begin to surface narratives, problem and resources available to person and family, prioritise problem and begin work of externalising  (ii) move to information-seeking stage to clarify how the problem works, out-of-session work is data collection or further work externalizing (such as naming and drawing)  (iii) begin data collection or review data and think strategy, out-of-session work requires a form of keeping data and strategy implementation  (iv) refine strategy based on data  (v) Reintegrate work back into the broader narrative, identify the work done and steps taken to identify problems | **Who**  People who use services (adolescents)  **Number**  10  **Age**  10-16 (13.7)8  **Gender Female:** 1 (10%)  **Autism diagnosis**  Y | **Adapted for autism:**  Modified in line with the discussion  by Cashin (2008).  **Theory informed:**  A foundation element of narrative therapy is the notion of individual  construction of meaning (Epston & White, 1995). Narrative therapy is aimed at surfacing alternate ways of seeing problems and how the  person is positioned in relation to the problem which in turn influences their ability to move forward in a less distressed way. It allows the therapist and individual to work on the isolation of a discrete problematic ‘chunk of behaviour rather than the amorphous problem-saturated situation’ | **Who delivers**  Qualiﬁed mental health nurse practitioner  **How**  NR  **For how long**  Five sessions of 1 hr duration every 2 weeks  **Where**  Southern Cross University Academic Health Centre | NA | Emotional/ behavioural problems  Psychological distress  Hopelessness  Stress |
| Social Skills  (Schiltz 2018) | **Aim:** Improve social skills, Increase social competence and potentially reduce depression  **Program for the Education and Enrichment of Relational Skills (PEERS®)** is a manualised, empirically supported social skills intervention for people with ASD. In addition to generalised social skills training, PEERS® includes specific sessions focused on handling teasing and bullying.  (i) Introduction and Conversational Skills I: Trading Information (ii) Conversational Skills II: Two-Way Conversations  (iii) Conversational Skills III: Electronic Communication  (iv) Choosing Appropriate Friends  (v) Appropriate Use of Humour  (vi) Peer Entry I: Entering a Conversation  (viii) Peer Entry II: Exiting a Conversation  (viii) Get-Togethers  (ix) Good Sportsmanship  (x) Rejection I: Teasing and Embarrassing Feedback  (xi) Rejection II: Bullying and Bad Reputations  (xii) Handling Disagreements  (xiii) Rumours and Gossip  (xiv) Graduation and Termination | **Who**  People who use services (adolescents and parents/caregivers)  **Number**  49  **Age**  Intervention: 13.25 (1.07) [12 – 15] Control: 13.52 (1.92) [11-16]  **Gender**  Women: Intervention: 8.3%, WL: 8.0%  **Autism diagnosis**  Y | **Adapted for autism:**  PEERS content, as well as the lesson format, was adapted from Children’s Friendship Training (CFT), an evidence-based parent-assisted social skills  curriculum  (Frankel and Myatt 2003). PEERS® modified the curriculum and methods of instruction, and added new modules, to be more applicable for  adolescents with Asperger’s Syndrome (AS) or  High Functioning Autism (HFA (Laugeson et al. 2009).  **Theory informed:**  Evidence suggests strong links between social difficulties, friendships, and depression in ASD, thus, interventions aimed at increasing social skills and, in turn, cultivating friendships, have the potential to ameliorate symptoms of depression | **Who delivers**  Trained graduate students in a clinical psychology PhD programme assisted with and co-led the PEERS adolescent and caregiver groups, and undergraduate students served as  coaches/assistants for the PEERS® groups.  **How**  Small group format involving  role-playing, modelling, coaching with performance feedback, and weekly socialisation assignments with consistent homework review  **For how long**  90-min sessions, delivered once a week, over the course of 14-weeks  **Where**  NR  **Completed**  Y | Wait list  Completed reports on the Children’s Depression Inventory (CDI) approximately 14 weeks apart and then participated  in PEERS® | Depression  Socialisation  Suicidal ideation |
| **Interventions: Training** | | | | | | |
| Training  (Cervantes et al., 2023a) | **Aim:** Improve clinician confidence and quality of care  Clinicians received mixed training. Training areas included:  (i) recognising and diagnosing ASD  (ii) recognising and diagnosing comorbid mental health disorders in youth with ASD  (iii) screening for and identifying suicide risk in youth with ASD  (iv) delivering intervention for the treatment of ASD symptoms  (v) delivering intervention for the treatment of comorbid mental health disorders in youth with ASD  (vi) managing and intervening on suicide risk in youth with ASD  (vii) coordinating care for patients with ASD across providers and service system | **Who**  Paediatric psychiatric clinicians in an emergency department (ED), who are mental health specialists and work with autistic patients, but are not necessarily specialised in ASD care  **Number**  16  **Age**  under the age of 40 - 14 (88%)  **Gender**  Women: 12 (75%)  **Autism diagnosis**  NA | **Adapted for autism:**  NA  **Theory informed:**  NR | **Who delivers**  Self-report  **How**  Completion of survey  **For how long**  NA  **Where**  NA  **Completed**  Y | NA | Clinician perceptions: suicide risk - Importance of addressing co-occurrence of ASD & STB  Clinician perceptions: suicide risk - Importance of adaptations to standard suicide risk and management practices  Clinician perceptions: suicide risk - Importance of adaptations to standard suicide risk and management practices  Clinician perceptions: suicide risk - Importance of screening for suicide for young people in general and autistic people specifically  Training log:  Training log:  Working with autistic people  Rates of STB  Providing suicide related care  Training impacting confidence |
| Training  (Cervantes et al., 2023b) | **Aim:** Encourage the implementation of routine suicide risk screening with autistic youth  Educational materials will be developed using data on barriers related to modifiable attitudes and behaviours of caregivers and clinicians from the first phase of the proposed study. These materials for clinicians will be disseminated via a webinar and posted on the Evidence-Based Treatment Dissemination Center’s website. The materials for caregivers will be disseminated through a webinar via the Regional Center for Autism Spectrum Disorders and available for download. Webinars will also be provided, and materials disseminated through the Autism Society of Central Virginia and Virginia Commonwealth University | **Who**  Specialised and non-specialised mental health clinicians and caregivers of autistic youth  **Number**  125 (Survey), 25 (interviews)  **Age**  NR  **Gender**  NR  **Autism diagnosis**  NA | **Adapted for autism:**  NR  **Theory informed:**  NR | **Who delivers**  Researchers in interviews and via webinar  **How**  Virtually and face-to=-face to interview participants  **For how long**  NA  **Where**  NR  **Completed**  In progress | NA | NA |
| Training  (Zoha 2022) | **Aim:** Increase safety in youth presenting to emergency departments  Safe Alternatives for Teens and Youths Acute (SAFETY-A) involves two educational videos about SAFETY-A  and a case simulation during the trainees’ rotation in the emergency service | **Who**  Trainees (child and adolescent psychiatry fellows (7), and  psychology interns (3))  **Number**  10  **Age**  NR  **Gender**  NR  **Autism diagnosis**  NA | **Adapted for autism:**  NR  **Theory informed:**  NR | **Who delivers**  NR  **How**  NR  **For how long**  NR  **Where**  Urban public hospital  **Completed**  Y | NA | NA |
| **Interventions: ECT/rTMS** | | | | | | |
| ECT  (Consoli 2013) | **Aim:** Alleviate self-injury/aggressive symptoms associated with severe and resistant psychiatric  disorders such as catatonia, severe mood disorders or schizophrenia  Anaesthesia for ECT was induced using intravenous propofol (10–170 mg, mean = 71.51 mg) and/or etomidate (2–12 mg, mean = 1.18 mg). Muscle relaxation was achieved with intravenous suxamethonium (25–70 mg, mean = 44.27 mg). ECT was given using a Thymatron-IV device that produces a brief pulse electric current (pulse width 1.0 ms, pulse duration 3.2 s) with a 30–70 Hz frequency. The electrical stimulus was applied using the standard bilateral electrode position with 10–30 % energy (0.75–0.92 A) | **Who**  People who use services (children)  **Number**  4  **Age**  13.8 [12-14]  **Gender**  Women: 2 (50%)  **Autism diagnosis**  N - only 2 PDD-NOS (pervasive developmental disorder-not otherwise specified (pervasive developmental) | **Adapted for autism:**  NR  **Theory informed:**  NR | **Who delivers**  NR  **How**  NR  **For how long**  average 19 ECT sessions [range 16–26] (one patient had maintenance ECT)  **Where**  Department of Child and Adolescent  Psychiatry at a university teaching hospital  **Completed**  Y | NA | Safety/tolerability  Efficacy: Aggression  Efficacy: Self-injurious behaviour/Aggression |
| Thea burst stimulation (rTMS)  NCT 2021 (Ameis 2024) | **Aim:** Reduce depression in young adults with autism spectrum disorder (ASD)  TBS is a newer form of rTMS shown to be non-inferior to conventional rTMS for depression with a similar safety profile.  Active bilateral theta burst stimulation. An X100 stimulator with a B65 A/P type coil (Magventure Inc.) will be used. The coil is positioned under MRI guidance using real-time neuro-navigation using Brainsight [x,y,z= -38, 44, 26(left), +38, 44, 26 (right). BL-TBS will be delivered at 90% RMT, corrected for scalp to cortex distance, to targeted left and right DLPFC sites, differing only in stimulation pattern and total number of pulses (triplet 50 Hz bursts, repeated at 200 msec (i.e., 5 Hz); right DLPFC (continuous TBS, cTBS): 120 seconds uninterrupted bursts (total of 600 pulses); left DLPFC (intermittent TBS, iTBS: two seconds on and eight seconds off; 600 pulses per session; total duration of 3 min 9 seconds/hemisphere). Device: Active Bilateral Theta Burst Stimulation | **Who**  People who use services (adults)  **Number**  80  **Age**  16-35 years  **Gender**  NR  **Autism diagnosis**  Y | **Adapted for autism:**  NR  **Theory informed:**  NA | **Who delivers**  Investigators  **How**  **For how long**  administered 5 days per week for 6 weeks- 30 active BL-TBS sessions  **Where**  NR  **Completed**  Trial in progress | Sham bilateral theta burst stimulation.  X100 stimulator with B65 A/P type coil (Magventure Inc.) used with active coil facing away from scalp, for sham stimulation. Coil positioned using MRI guidance+real-time neuro-navigation. To reproduce nociceptive qualities of stimulation, B65-type stimulation coil - sham side - includes built-in electrical stimulator in coil connector which "fires" synchronous electrical pulse with TMS stimulus through electrodes on forehead/near stimulation area, to generate auditory and somatosensory stimuli. Device: Sham Bilateral Theta Burst Stimulation | Efficacy: Depression  Efficacy: Suicidal ideation |
| AASP=Autism Adapted Safety Plans, ASD=Autism Spectrum Disorder, CBT=Cognitive Behavioural Therapy, CSQ=Client Satisfaction Questionnaire, DBT=Dialectical Behaviour Therapy, DLPFC=Dorsolateral Prefrontal Cortex, ECT=Electro-Convulsive Therapy, ESP=Emotional Support Plan, HFA=High Functioning Autism, MCAS-R=Mental Health Crisis Assessment Scale-Revised, NA=Not Applicable, NR=Not Reported, PDD-NOS=Pervasive Developmental Disorder-Not Otherwise Specified, QOL=Quality of Life, rTMS=Repetitive Transcranial Magnetic Stimulation, SITBI=Self-injurious Thoughts and Behaviours Inventory, SP=Safety Plan, SPI(A)=Safety Planning Intervention (for individuals with Autism), STB=Suicidal Thoughts and Behaviour, SUS=System Useability Scale, TAU=Treatment as Usual, VEQ=Vulnerabilities Experience Quotient, WL=Waiting List, Y=Yes, YCMAP=Youth Culturally Adapted Manual Assisted Psychological Intervention | | | | | | |

**Supplementary File 9: Studies evaluating suicide-screening procedures**

| **Name of screening tool: Tool aim (First author, date)** | **Domains covered by tool: Domain of interest** | **N items specific to suicidality/ Total number of items** | **Who completed (N, % female): Who administered** | **Setting: How screening implemented** | **Outcomes measured** |
| --- | --- | --- | --- | --- | --- |
| **Universal screening tools** | | | | | |
| **Suicide Behaviours Questionnaire-Revised (SBQ-R):** Assess risk of suicide  (Cassidy 2020) | 4 suicide-related questions: All | 4/4 (lifetime suicidal behaviour, suicide ideation over the past 12 months, threat of suicide attempt, likelihood of suicidal behaviour in the future) | People who use services (15, 47% f): Online administration | NR: Online administration. Sub-sample completed in room and provided qualitative feedback with "think aloud" approach | Qualitative themes |
| **Kiddie-Computerized Adaptive Test (K-CAT) at & K-CAT-Suicide Scale (K-CAT-SS**): Measure suicide risk in children and adolescents (not validated for use with autistic youth (Cervantes et al., 2024a) | Eight scales on the K-CAT (anxiety, depression, mania, attention-deficit/hyperactivity disorder, oppositional defiant disorder, conduct disorder, substance use disorder, suicide) & the K-CAT-SS is one of the eight scales: K-CAT-Suicide Scale (K-CAT-SS) | K-CAT-SS: 64 items | Young people (5, 60% f) and caregivers (6, 83.3% f) completed K-CAT: Self-report | Intended for use in ED: Electronically administered. Time: independently <2mins, complete battery <10 mins. Youth/caregiver version of full K-CAT; only youth version of K-CAT-SS exists | Qualitative themes |
| **MH Crisis Assessment Scale-Revised (MCAS-R):** Identify psychiatric crisis by evaluating dangerousness of child behaviour and caregivers’ perceived ability to manage child’s behaviour. Also identifies which particular challenging behaviours that can result in crisis (Kalb et al., 2022) | Section 1: Severity of 13 MH behaviours (Self-injury, physical/verbal aggression, elopement, property destruction, dangerous impulsivity, depression, suicidal thoughts/behaviours, tantrums, oppositional, psychosis, sudden, worrisome change, pica): Self-injury, suicidal thoughts/behaviour | 2 | Caregiver (404, 14% mothers): Self-report^b^ | Telehealth from 2 outpatient ASD specialty centres: Electronic link to MCAS-R. Parent provides demographic and clinical information about child and family. MCAS-R: 23-item, 5–10 min to complete. 3 sections+ 3-month reporting window. Section 1: severity of 13 MH behaviours. If none rated moderate/severe, remainder of MCAS-R not completed. If 1 or more behaviour moderate/severe complete Section 2: select up to three 13 behaviours that could cause greatest harm to child. Section 3: 8 questions about dangerousness of behaviour(s)+3 questions on parent ability to manage child’s behaviour(s), based on 5-point Likert scale. Electronic administration depended on site. Psychiatric clinic: MCAS-R emailed to family via a survey link 1wk before appointment. Email/telephone reminder 3 days before appointment, with additional reminder by study coordinator All caregivers provided crisis contact information if immediate concerns. Caregivers of youth positive for crisis risk: offered emergency SW evaluation. Behavioural clinic: clinicians provided MCAS-R link for parent to complete during virtual therapy appointments. Both clinics: clinicians received e-mail providing results to incorporate into clinical evaluation | Feasibility, Internal/ External validity, Parent/Clinician satisfaction |
| **Ask Suicide Questions** | | | | | |
| **Kiddie-Computerized Adaptive Test - Suicide Scale (K-CAT-SS) :** Measures suicide risk in children and adolescents (Cervantes et al., 2024c) | Suicidal thoughts and behaviours, and suicide risk: NA | 10/10 and 65 items from Depression scale of K-CAT predictive of suicide risk | Young people (183, 69.4% f) and carers (146, 72.6% mothers) : Research assistants^a^ | Intended for use in ED: Electronically administered electronically on tablet. Not validated for use with autistic youth | Participant demographics, Mood, Anxiety, Risk, Suicidality, Discrepancy between tools |
| **Ask Suicide-Screening Questions (ASQ)** (comparison of two tools): Measures youth suicide risk (Cervantes et al., 2024c) | Suicide risk : NA | 5 of 5 | See above | Already in use in ED: Electronically administered electronically on tablet | See above |
| **Suicidal ideation questionnaire - child and parent version**: Establish risk of suicide (Horowitz 2018) | NR: NR | All (assumed) | Child (14, NR) or caregivers (NR): NR | Community health centre: NR | Current and historical suicidality |
| **Ask Suicide-Screening Questionnaire (ASQ)**: Identify children at risk of suicide (Kalari 2022) | Suicide: NR | All (assumed) | Child (assumed)(393 with ASD, 52.1% f): NR | Urban paediatric ED: NR | Demographics, Presenting concern, Suicidality |
| **Columbia-Suicide Severity Rating Scale:** Assess suicide risk and guide safety planning (Rybczynski 2022) | Suicide ideation and behaviour: NA | 10 of 10 | Children 8-12 yrs with parent/ guardian present (NR): Registered Nurse | Tertiary paediatric NDD and rehabilitation centre: Interdisciplinary task force (PM, CP, nursing, SW, psych.) designed and implemented suicide risk screening program for all medical clinics. All involved clinical staff received education in suicide prevention, diagnosis and management of SI and screening protocol. Training sessions led by study team members with experience in suicide prevention research and education. Standardized tools to assess suicide risk/guide safety plans presented (e.g. CBSS and ASQ toolkit’s BSSA). Use of these tools, particularly ASQ BSSA, encouraged, but not mandatory, for patients positive for increased suicide risk. Format: Lectures/written. Parents could answer questions on behalf of child. Children>12 years screened without parent/guardian present with parental approval. Patients, parent/guardians could decline participation in screening. RN completed screening during appointment triage. Before screening, nurses used standard script (screening purpose, normalized procedure, confidentiality). If concerns RE: self-harm or safety, parents notified immediately and included in follow-up planning. ASQ administered verbatim verbally. No written material used for patient self-report. If patient positive screening, medical staff members notified. Further suicide risk assessment performed by physician, NP or MHP (clinical SW or psychologist). Management/ follow-up treatment plans based on suicide risk assessment. Options for follow-up: additional visits with treating physician or NP, referral to new outpatient MH therapy services or psychiatry, follow-up with current MH therapist or psychiatrist, referral to local ED for evaluation or acute psychiatric care (intensive day treatment or inpatient) | Identification suicidal intent |
| **Ask Suicide-Screening Questions:** Assess suicide risk and establish active suicidal intent. Guide safety planning (Rybczynski 2022) | Suicide ideation and behaviour: NA | 4 of 4 | See above. ASQ does not have parent version | See above. Use of these tools, in particular the ASQ BSSA, was encouraged, but not mandatory, for patients who screened positive for increased suicide risk | See above |
| **Ask Suicide-Screening Questionnaire (ASQ): I**dentify children at risk of suicide (Vasa 2017) | Suicide: NR | All (assumed) | Child (assumed) (104, NR): NR | Paediatric ED: Part of standard care in triage of ED | Demographics, Suicidality |
| ^a^% Female of young people based on sex; ^b^% mother based on N where relationship to child data provided. ASD=Autistic Spectrum Disorder, ASQ=Ask Suicide Screening Questions, BSSA=Brief Suicide Safety Assessment, CBSS=Columbia-Suicide Severity Rating Scale, CP=Child Psychiatry, ED=Emergency Department, F=Female, K-CAT=Kiddie-Computerized Adaptive Test, K-CAT-SS=Kiddie-Computerized Adaptive Test Suicide Scale, MCAS-R=Mental Health Crisis Assessment Scale-Revised, MH=Mental Health, MHP=Mental Health Professional N=Number, NDD=Neurodevelopmental Disabilities, NP=Nurse Practitioner, NR=Not Reported, PM=Paediatric Medicine, RN=Registered Nurse, SBQ-R=Suicide Behaviours Questionnaire-Revised, SW=Social Worker. Blue shaded cells=different tools used within same study | | | | | |
